# Supplementary material for: Rationale and population-based prospective cohort protocol for the disadvantaged populations at risk of decline in eGFR (CO-DEGREE)
Source: BMJ Open. 2019 Sep 24;9(9):e031169. doi: 10.1136/bmjopen-2019-031169 (PMC6773312; doi:10.1136/bmjopen-2019-031169)
Supplement: Supplementary data [file bmjopen-2019-031169supp001.pdf]

CO-DEGREE: baseline questionnaire

PASTE THE ID LEVEL HERE

Participant Identification Number └─┴─┴─┘ └─┴─┴─┘ └─┴─┴─┘

CO-DEGREE Basic Core Questionnaire

	Response	Code
Study site ID	└─┴─┴─┘	I1
Interviewer ID	└─┴─┴─┘	I2
Study visit number	└─┴─┴─┘	I3
Date of completion of the instrument	└─┴─┘ └─┴─┘ └─┴─┴─┴─┘<br>dd mm year	I4

Consent, Interview Language and Name	Response	Code
Consent has been read and obtained	Yes 1<br>No 2 If NO, END	I5
Interview Language *[Insert Language]*	English 1<br>*[Add others]* 2<br>*[Add others]* 3<br>*[Add others]* 4	I6
Time of interview<br>(24 hour clock)	└─┴─┘ : └─┴─┘<br>hrs mins	I7
Family Surname		I8
First Name		I9
Address:		
Additional Information that may be helpful		
Contact phone number where possible		I10

Gonzalez-Quiroz M, *et al. BMJ Open* 2019; 9:e031169. doi: 10.1136/bmjopen-2019-031169

CO-DEGREE: baseline questionnaire

PASTE THE ID LEVEL HERE

Participant Identification Number |_|_|_| |_|_|_| |_|_|_|

CO-DEGREE basic core clinical and anthropometry measurements

Question	Response	Code
Ambient Temperature (at time of examination measured in shade)	|_|_|_| °C	Temp
**Blood Pressure**		
Question	Response	Code
Interviewer ID	|_|_|_|	M1
Device ID for blood pressure	|_|_|_|	M2
Cuff size used	Small 1<br>Medium 2<br>Large 3	M3
Reading 1	Systolic (mmHg) |_|_|_|	M4a
	Diastolic (mmHg) |_|_|_|	M4b
	Heart rate |_|_|_|	M4c
Reading 2	Systolic (mmHg) |_|_|_|	M5a
	Diastolic (mmHg) |_|_|_|	M5b
	Heart rate |_|_|_|	M5c
Reading 3	Systolic (mmHg) |_|_|_|	M6a
	Diastolic (mmHg) |_|_|_|	M6b
	Heart rate |_|_|_|	M6c
During the past two weeks, have you been treated for raised blood pressure with drugs (medication) prescribed by a doctor or other health worker?	Yes 1<br>No 2	M7
**Height, and Weight**		
For women: Are you pregnant?	Yes 1 if yes should be excluded<br>No 2	M8
Have you eaten yet today?	Yes 1<br>No 2	M9
Interviewer ID	|_|_|_|	M10
Height	in Centimetres (cm) |_|_|_|	M11
Weight<br>*If too large for scale 666.6*	in Kilograms (kg) |_|_|_|	M12

Gonzalez-Quiroz M, *et al. BMJ Open* 2019; 9:e031169. doi: 10.1136/bmjopen-2019-031169

CO-DEGREE: baseline questionnaire

PASTE THE ID LEVEL HERE

Participant Identification Number |_|_|_|_| |_|_|_|_| |_|_|_|_|

CO-DEGREE basic core questionnaire (adapted from DEGREE PROTOCOL)

Question	Response	Code
Sex (*Record Male / Female as observed)*	Male 1<br>Female 2	C1
What is your date of birth?<br>*Don't Know 77 77 7777*	dd mm year	C2
How old are you?	Years |_|_|_|	C3
In total, how many years have you spent at school and in full-time study (excluding pre-school)?	Years |_|_|_|	C4
What is your *[insert relevant ethnic group / racial group / cultural subgroup / others]* background?	*[Locally defined]* 1<br>*[Locally defined]* 2<br>*[Locally defined]* 3<br>Refused 88	C6
Which of the following best describes your main work status over the past 12 months?	Government employee 1<br>Non-government employee 2<br>Self-employed 3<br>Non-paid 4<br>Student 5<br>Homemaker 6<br>Retired 7<br>Unemployed (able to work) 8<br>Unemployed (unable to work) 9<br>Unpaid domestic 10<br>Refused 88	C8
If you are working what is your main occupation [FREE TEXT]:		OCCTXT
What task do you perform? [FREE TEXT]		TASKTXT
How many years have you been working in your current job?	Years |_|_|_|	C9
How many hours do you work daily?	Hours |_|_|_|	C10
Where do you work mostly?	Indoors 1<br>Outdoors 2<br>Both 3	C11
Do you take work breaks in shade?	Yes 1<br>No 2	C12
Do you work in a very hot working environment?	Seldom or never 1<br>Few times 2<br>Regularly 3<br>Frequently 4<br>Always or almost always 5	C13
How much physical effort did you do at work?	Slight effort 1<br>Moderate effort 2<br>Hard effort 3<br>Very hard effort 4	C14
Do you have experience of migrant work?<br>[Defined as staying far from home for seasonal work]	Yes *1*<br>No 2	MIGR
Can you give an estimate of the monthly household income if I read some options to you? Is it<br>*[INSERT QUINTILE VALUES IN LOCAL CURRENCY]*<br><br>*(READ OPTIONS)*	≤ Quintile (Q) 1 1<br>More than Q 1, ≤ Q 2 2<br>More than Q 2, ≤ Q 3 3<br>More than Q 3, ≤ Q 4 4<br>More than Q 4 5<br>Don't Know 77<br>Refused 88	C15

CO-DEGREE: baseline questionnaire

PASTE THE ID LEVEL HERE

Participant Identification Number |__|__|__|__| |__|__|__|__| |__|__|__|__|

During the past 12 months, how frequently have you had at least one standard alcoholic drink?<br><br>*(READ RESPONSES, USE SHOWCARD)*	Daily 1<br>5-6 days per week 2<br>3-4 days per week 3<br>1-2 days per week 4<br>1-3 days per month 5<br>Less than once a month 6<br>Not at all 7<br>Refused 88	A4
Do you currently smoke any tobacco products, such as cigarettes, cigars or pipes? *(USE SHOWCARD)*	Yes 1<br>No 2	*T1*
In a typical week, on how many days do you eat MEAT *(USE SHOWCARD)*	Number of days<br>Don't Know 77 |__|__|	*D1*
Does your work involve vigorous-intensity activity that causes large increases in breathing or heart rate like *[carrying or lifting heavy loads, digging or construction work]* for at least 10 minutes continuously? *(OR USE SHOWCARD)*	Yes *1*<br>No 2	*P1*
Have you ever been told by a doctor or other health worker that you have raised blood pressure or hypertension?	Yes 1<br>No 2	H2a
Have you ever been told by a doctor or other health worker that you have raised blood sugar or diabetes?	Yes 1<br>No 2	H7a
Have you used agrichemicals?	Yes 1<br>No 2	L1
Did you mix, apply or both?	Mix 1<br>Apply 2<br>Both 3	L2
Have you been diagnosed with?	Dengue 1<br>Chikungunya 2<br>Zika 3<br>Malaria 4	L3
**Renal Protocol**		
Has a doctor diagnosed you with kidney disease?	No 1 go to question KI3<br>Yes 2 go to question KI2	KI1
Have you been told you have one of these kidney disease?	Glomerulonephritis 1<br>Congenital abnormality of the 2<br>kidneys 3<br>Polycystic kidney disease 4<br>Diabetic kidney disease 5<br>[locally defined] 6<br>[locally defined] 7<br>[locally defined] 8	KI2
Have you been told you have ever been told you have one of these diseases?	Tuberculosis 1<br>HIV 2<br>Hepatitis B 3<br>Hepatitis C 4<br>Schistosomiasis 5<br>Leptospirosis 6<br>[locally defined] 7<br>[locally defined] 8	KI3
Do you take herbal or traditional remedies?	No 1<br>Yes 2	KI4
Do you take regular prescribed medications?	No 1 go to question KI 9<br>Yes 2 2 go to questions below	KI5
Do you take medication for diabetes?	No 1<br>Yes 2	KI6
Do you take medication against HIV or hepatitis?	No 1<br>Yes 2	KI7
Do you take medication for tuberculosis?	No 1<br>Yes 2	KI8
Have you used painkillers most days for more than several months?<br>[Use Showcard with locally available medications]?	No 1<br>Yes 2	KI9

Gonzalez-Quiroz M, *et al. BMJ Open* 2019; 9:e031169. doi: 10.1136/bmjopen-2019-031169

CO-DEGREE: baseline questionnaire

PASTE THE ID LEVEL HERE

Participant Identification Number |_|_|_| |_|_|_| |_|_|_|

DEGREE study core lab measurements

Question	Response		Code
Blood sampling Investigator ID		|_|_|_|	B2
Time of day blood specimen taken (24 hour clock)	Hours : minutes	|_|_| : |_|_| hrs mins	B4
Creatinine measurement Technician ID		|_|_|_|	CR1
Creatinine measurement Device ID		|_|_|_|	CR2
Serum Creatinine	*to first decimal place if in mg/dL*	|_|_|_|	CR3
Serum Creatinine Units	mg/dL	1	CR4
	μMol/L	2	
Urine sampling Investigator ID		|_|_|_|	UR1
Urinalysis Device ID		|_|_|_|	UR2
Urine Glucose	Negative	1	UR3
	100mg/dL	2	
	250mg/dL	3	
	500mg/dL	4	
	1000mg/dL	5	
	>2000mg/dL	6	
Urine Specific Gravity	1.000	1	UR4
	1.005	2	
	1.010	3	
	1.015	4	
	1.020	5	
	1.025	6	
	1.030	7	
Urinalysis Blood	Negative	1	UR5
	Non-haemolysed trace	2	
	Non-haemolysed moderate	3	
	Haemolysed trace	4	
	Small (+)	5	
	Moderate (++)	6	
	Large (+++)	7	
Urine pH	5.0	1	UR6
	6.0	2	
	6.5	3	
	7.0	4	
	7.5	5	
	8.0	6	
	8.5	7	
Urinalysis Protein	Negative	1	UR7
	Trace	2	
	30mg/dL (+)	3	
	100mg/dL (++)	4	
	300mg/dL (+++)	5	
	>2000mg/dL	6	
Urinalysis Nitrite	Negative	1	UR8
	Positive	2	
Urinalysis Leucocytes	Negative	1	UR9
	Trace	2	
	Small (+)	3	
	Moderate (++)	4	
	Large (+++)	5	
