# Supplementary material for: Rationale and population-based prospective cohort protocol for the disadvantaged populations at risk of decline in eGFR (CO-DEGREE)
Source: BMJ Open. 2019 Sep 24;9(9):e031169. doi: 10.1136/bmjopen-2019-031169 (PMC6773312; doi:10.1136/bmjopen-2019-031169)
Supplement: Author's manuscript [file bmjopen-2019-031169.draft_revisions.pdf]

BMJ Open is committed to open peer review. As part of this commitment we make the peer review history of every article we publish publicly available.

When an article is published we post the peer reviewers' comments and the authors' responses online. We also post the versions of the paper that were used during peer review. These are the versions that the peer review comments apply to.

The versions of the paper that follow are the versions that were submitted during the peer review process. They are not the versions of record or the final published versions. They should not be cited or distributed as the published version of this manuscript.

BMJ Open is an open access journal and the full, final, typeset and author-corrected version of record of the manuscript is available on our site with no access controls, subscription charges or pay-per-view fees (http://bmjopen.bmj.com).

If you have any questions on BMJ Open's open peer review process please email info.bmjopen@bmj.com

BMJ Open

Rationale and Population-based prospective cohort protocol for the Disadvantaged Populations at Risk of Decline in eGFR (CO-DEGREE)

Journal:	*BMJ Open*
Manuscript ID	bmjopen-2019-031169
Article Type:	Protocol
Date Submitted by the Author:	19-Apr-2019

Complete List of Authors:	Gonzalez-Quiroz, Marvin ; National Autonomous University of Nicaragua, Research Centre on Health, Work and Environment<br>Nitsch, Dorothea; LSHTM<br>Hamilton, Sophie ; Imperial College London, School of Public Health, Faculty of Medicine<br>O'Callaghan Gordo, Cristina; Instituto de Salud Global Barcelona, Campus Mar<br>Glaser, Jason; La Isla Network<br>Correa-Rotter, Ricardo; National Medical Science and Nutrition Institute Salvador Zubirán, Dept. Nephrology and Mineral Metabolism<br>Jakobsson, Kristina; Goteborgs Universitet,<br>Singh, Ajay; Brigham and Women's Hospital and Harvard medical School<br>Gunawardena, Nalika; World Health Organization Country Office<br>Levin, Adeera; University of British Columbia, Medicine<br>Remuzzi, Giuseppe; IRCCS - Istituto di Ricerche Farmacologiche Mario Negri, Centro Anna Maria Astori, Science and Technology Park Kilometro Rosso<br>Caplin, Ben; University College London Medical School, Centre for Nephrology,<br>Pearce, Neil; London School of Hygiene and Tropical Medicine, Medical Statistics

Keywords:	Prospective cohort study, Chronic kidney disease of unknown aetiology, Generic cohort protocol, Decline in kidney function

SCHOLARONE™
Manuscripts

Rationale and Population-based prospective cohort protocol for the Disadvantaged Populations at Risk of Decline in eGFR (CO-DEGREE)

Marvin Gonzalez-Quiroz, Dorothea Nitsch, Sophie Hamilton, Cristina O´Callaghan-Gordo, Jason Glaser, Ricardo Correa-Rotter, Kristina Jakobsson, Ajay Singh, Nalika Gunawardena, Adeera Levin, Giuseppe Remuzzi, Ben Caplin, Neil Pearce, on behalf of the DEGREE Study Steering Committee

Marvin González-Quiroz, MD, PhD. Research Centre on Health, Work and Environment (CISTA), National Autonomous University of Nicaragua at León (UNAN-León), León, Nicaragua. Centre for Nephrology, University College London, London, UK. Department of Non-Communicable Disease Epidemiology, London School of Hygiene and Tropical Medicine, London, UK. m.quiroz@ucl.ac.uk or marvin99_00@yahoo.es ORCID ID: 0000-0002-0093-6357

Dorothea Nitsch, *Dr.med.* Department of Non-Communicable Disease Epidemiology, London School of Hygiene and Tropical Medicine, London, UK. Dorothea.Nitsch@lshtm.ac.uk

Sophie Hamilton, MSc. School of Public Health, Faculty of Medicine at Imperial College London, London, UK. s.hamilton16@ic.ac.uk

Cristina O´Callaghan-Gordo, PhD. ISGlobal, Barcelona, Spain; Universitat Pompeu Fabra (UPF), Barcelona, Spain; CIBER Epidemiología y Salud Pública (CIBERESP), Madrid, Spain; cristina.ocallaghan@isglobal.org

Jason Glaser, BSc. La Isla Network, Washington DC, USA. jason@laislanetwork.org

Ricardo Correa-Rotter, MD. Department of Nephrology and Mineral Metabolism, National Medical Science and Nutrition Institute Salvador Zubirán, Mexico, DF. correarotter@gmail.com

Kristina Jakobsson, MD, PhD. Department of Public Health and Community Medicine, Institute of Medicine, Sahlgrenska Academy, University of Gothenburg, Gothenburg, Sweden. Occupational and Environmental Medicine, Sahlgrenska University Hospital, Region Västra Götaland, Gothenburg, Sweden. kristina.jakobsson@amm.gu.se

For peer review only - http://bmjopen.bmj.com/site/about/guidelines.xhtml

Ajay Singh, MD. Brigham and Women's Hospital and Harvard medical School, Boston, Massachusetts, USA. Ajay_Singh@hms.harvard.edu

Nalika Gunawardena, MD. World Health Organization Country Office, Colombo, Sri Lanka. gunawardenan@who.int

Levin Adeera, MD. Division of Nephrology UBC, University of British Columbia, ALevin@providencehealth.bc.ca

Giuseppe Remuzzi, *MD.* Instituto di Ricerche Farmacologiche Mario Negri IRCCS, Milan, Italy. giuseppe.remuzzi@marionegri.it

Ben Caplin*, *PhD.* Centre for Nephrology, University College London Medical School, London, UK. b.caplin@ucl.ac.uk

Neil Pearce*, *PhD.* Department of Medical Statistics and Non-Communicable Disease Epidemiology, London School of Hygiene and Tropical Medicine, London, UK, Centre for Global NCDs, London School of Hygiene and Tropical Medicine, London, UK. Neil.Pearce@lshtm.ac.uk

**Equal contribution*

Corresponding author: Marvin Gonzalez-Quiroz

Research Centre on Health, Work and Environment (CISTA), National Autonomous University of Nicaragua at León (UNAN-León), León, Nicaragua

Address: Campus Médico, Facultad de Ciencias Médica, edificio C, León, Nicaragua

Tel: +505 89368376

Email: m.quiroz@ucl.ac.uk or *marvin99_00@yahoo.es*

This **original article** has been seen and approved by all authors listed above and is not under consideration for publication elsewhere.

Word count for Abstract: 297

For peer review only - http://bmjopen.bmj.com/site/about/guidelines.xhtml

Word count for text: 4435

Total word count including tables and figures: 6882

For peer review only

For peer review only - http://bmjopen.bmj.com/site/about/guidelines.xhtml

Abstract

Introduction

A recently recognised form of chronic kidney disease (CKD) of unknown origin (CKDu) is afflicting communities, mostly in rural areas in several regions of the world. Prevalence studies are being conducted in a number of countries, using a standardised protocol, to estimate the distribution of estimated glomerular filtration rate (eGFR), and thus identify communities with a high prevalence of reduced GFR. In this paper, we propose a standardized minimum protocol for cohort studies in high-risk communities aimed at investigating the incidence of, and risk-factors for, early kidney dysfunction.

Methods and analysis

This generic cohort protocol provides the information to establish a prospective population-based cohort study in low-income settings with a high prevalence of CKDu. This involves a baseline survey that included key elements from the DEGREE survey (e.g., using the previously published DEGREE methodology) of a population-representative sample, and subsequent follow-up visits in young adults (without a pre-existing diagnosis of CKD (eGFR<60 mL/min/1.73m^2), proteinuria, or risk factors for CKD at baseline) over several years. Each visit involves a core questionnaire, collection and storage of biological samples. Local capacity to measure serum creatinine (sCr) will be required so that immediate feedback on kidney function can be provided to participants. After completion of follow-up, repeat measures of creatinine should be conducted in a central laboratory, using reference standards traceable to isotope dilution mass

For peer review only - http://bmjopen.bmj.com/site/about/guidelines.xhtml

spectrometry (IDMS) quality control material to quantify the main outcome of eGFR decline over-time, alongside a description of the early evolution of disease and risk factors for eGFR decline.

Ethics and dissemination

Ethical approval will be obtained by local researchers, and participants will provide informed consent before the study commences. Participants will typically receive feedback and advice on their laboratory results, and referral to a local health system where appropriate.

Trial registration number: Not applicable

For peer review only - http://bmjopen.bmj.com/site/about/guidelines.xhtml

Strengths and limitations of this study

- *We propose a prospective generic cohort protocol for populations affected by CKDu in which the sampling frame consists of the entire at-risk population.*
- *Serial eGFR measurements in an apparently healthy population will allow the description of the evolution of disease and reduce problems associated with recall bias and reverse causation when assessing potential risk factors.*
- *Samples will be analysed in a single batch at the end of the study to minimize time-dependent measurement errors.*
- *A biobank is expected to be created in each centre to store biological samples for future analyses.*
- *The use of a standardised protocol will allow for regional and international comparisons.*

For peer review only

Introduction

A mysterious form of chronic kidney disease (CKD) is afflicting young adults, mostly in rural communities in a number of low- and middle-income countries.[1-10] This disease has been termed CKD of undetermined cause (CKDu). Several definitions for CKDu exist; the criteria typically include demonstration of renal damage using biomarkers in the absence of diabetes, severe hypertension or evidence of alternative renal diagnoses.[11-14]. This syndrome has caused thousands of deaths and reduced the life expectancy among young adults in Mesoamerica, South Asia, and possibly in other tropical/subtropical regions of the world.[7, 15-19] The cause(s) of CKDu are not yet established, but proposed aetiologies include recurrent dehydration/heat stress, pesticides, infections, and heavy metals.[1, 20-22] In addition, there is no evidence that these forms of CKDu have a unified causality or are due to different aetiologies in diverse parts of the world.

Although a broad range of cross-sectional studies investigating prevalence of CKDu have been conducted in Mesoamerica, South Asia, and other regions of the world,[1-7, 9, 17], these have generally not used standardised methodology, and therefore do not allow for valid international comparisons. A recently published standardised protocol (the Disadvantaged Populations eGFR Epidemiology Study (DEGREE) protocol) for estimating the population distribution of glomerular filtration rate (eGFR), has addressed this concern, and is being used in communities suspected to have a high prevalence of reduced eGFR. The DEGREE protocol makes it possible to undertake comparisons internationally, by mandating a population-representative sample and standardised

For peer review only - http://bmjopen.bmj.com/site/about/guidelines.xhtml

collection of information on sociodemographic factors, occupational and environmental exposures, body composition and kidney function.[23] To date, studies using the DEGREE methodology have been conducted in four countries (Peru, Sri Lanka, India, Malawi), with a number of future projects in preparation or in progress.[17]

A recent meta-analysis highlighted the lack of robust studies that have considered risk factors for early kidney damage in CKDu.[24] This is of key importance as those with even apparently mildly-damaged kidneys (e.g. a borderline elevated serum creatinine but no renal reserve) may experience progressive renal decline in response to a wide-range of exacerbating insults (e.g. episodes of dehydration/heat stress or use of nephrotoxic medication or other nephrotoxic exposures) making identification of causal associations challenging in those with existing kidney damage. Based on our experience[25, 26] we propose a generic cohort protocol to characterise the decline in kidney function over time and conduct aetiological research in those without pre-existing CKD/risk-factors at baseline but at risk of CKDu. Our focus is on conducting such cohort studies in populations which are at high risk for CKDu i.e., that have previously been classified as such by surveys based on cross-sectional eGFR measurements. In general, this work would follow on from a study using the DEGREE protocol, and hence we will use the term 'CO-DEGREE' (cohorts based on the DEGREE study) for such studies. Indeed, in some situations, a DEGREE survey may form the 'baseline', with a subgroup of DEGREE survey participants then being selected for follow-up based on age, a single measurement of eGFR ≥60 mL/min/1.73 m^2 (accepting that this is likely a conservative cut-off for pre-existing kidney dysfunction), and without clinical diagnosis or history of hypertension,

For peer review only - http://bmjopen.bmj.com/site/about/guidelines.xhtml

diabetes mellitus, obesity, or other known risk factor that could potentially explain CKD. However, the standardised protocol we propose here can also be used as a ‘stand-alone’ study design in any well-defined study group, without requiring that a DEGREE survey is conducted first.

We are already conducting such a cohort study in Nicaragua,[25, 26] and have had many challenges to address, including: (i) community engagement, awareness of conditions, political unrest and ethics; (ii) follow-up over time (frequency and minimising loss to follow-up); (iii) fieldwork and laboratory standards to ensure decline is detected; and (iv) regular feedback information on study progress. We will draw on our experience in Nicaragua in presenting both the generic CO-DEGREE protocol, as well as observations on the practical issues involved in conducting such studies in a particular population.

Objectives

Studies using this generic cohort protocol, and contributing to the wider DEGREE collaboration, will aim to:

1. Investigate the evolution of, and risk factors for, kidney function decline over time among populations at risk of CKDu.
2. Compare the evolution, and risk factors for kidney function decline, in different populations and regions at risk of CKDu.
3. Establish a framework for international collaboration and promote a network for future work on the causality of CKDu.

For peer review only - http://bmjopen.bmj.com/site/about/guidelines.xhtml

Rationale for a cohort study of decline in eGFR

A representative sample of those at-risk

Population-based cohort studies have several advantages:[27] Firstly this type of study allows the recruitment of a representative sample of the at-risk population, e.g., it will include workers from a variety of occupations (including unemployed) at the community level. Assuming that the study sample is randomly selected from the entire at-risk population based on a community census, and there are no substantial problems with non-response, these studies are unlikely to be affected by significant selection bias. Furthermore, in contrast to studies conducted solely in an occupational setting, differential loss to follow up is likely to be less problematic, particularly if workers are screened for kidney disease within that setting and potentially denied further work.

Like all prospective cohort studies, to ensure the entire population is 'at-risk', those with the outcome at baseline should be excluded, although it is recognised that investigators may wish to follow-up those with eGFR <60 mL/min/1.73 m^2 and those with established risk factors for CKD for other purposes (see below).

One general disadvantage of population-based studies is that this approach typically requires large sample sizes and long-term follow-up if disease is not highly prevalent. However, the focus of CO-DEGREE is on conducting studies in population with a high prevalence of CKDu (see below).[25, 27]

Handling reverse causation and recall bias

For peer review only - http://bmjopen.bmj.com/site/about/guidelines.xhtml

The problem of reverse-causation (e.g., modification of behaviour or work tasks in response to the diagnosis of renal impairment) can be minimised in a cohort study by focusing on people without pre-existing disease, and then following these initially apparently 'healthy' participants over time. Similarly, a cohort approach unlike cross-sectional studies is less prone to recall bias regarding previous exposures.

Measuring kidney function

Quantification of kidney function is most easily undertaken by determining serum creatinine (sCr) concentration, which is relatively easy and cheap to measure, and then calculating the eGFR. A case of CKDu is typically defined by an eGFR <60 mL/min/1.73 m^2 (sustained for at least 3 months to confirm chronicity) in the absence of known causes of kidney disease. However, this dichotomous definition has weaknesses in studies exploring the causation of CKDu, as it is well established that substantial damage may have already occurred at the histological level before serum biomarkers of renal dysfunction become abnormal (and other markers such as proteinuria are often absent in this disease). Furthermore, repeat measures after 3-months are not always performed in cross-sectional surveys, and sCr levels are modified by multiple non-renal factors such as: high animal protein-intake, strenuous exercise, changes in plasma volume, body mass index (BMI), sex, age, ethnicity, and some drugs;[(28)] thus, cross-sectional studies examining associations with reduced eGFR based on a single sCr measurement may be prone to a significant degree of misclassification, especially in smaller studies. Notably, the accuracy of sCr determinations is also an inherent problem (see further below). In

For peer review only - http://bmjopen.bmj.com/site/about/guidelines.xhtml

addition, the CKD-EPI or MDRD equations used to calculate eGFR from sCr,[28] have not been validated in many populations reported to be suffering CKDu,[29] potentially further increasing misclassification bias in cross-sectional studies.

Alternative approaches based on serial eGFR measurements in the same person over time render between-person variation less problematic. If estimated across a period of time using multiple measures with sustained preanalytical and analytical quality, this will also reduce the influence of the within-person factors that are not directly related to kidney damage. In summary, an approach utilising serial eGFR measures substantially improves the potential to identify risk/causal factors for CKDu as well as allowing the description of the evolution of disease.

Core protocol

Study design

This is a prospective cohort study protocol for studying decline in kidney function over time in populations with high reported prevalence of CKDu, primarily in low- and middle-income countries (LMICs). We consider the following study design issues: (i) population sampling strategy, and follow-up interval (ii) questionnaire development and delivery, (iii) clinical measurements and biosampling, and (iv) data management and reporting.[25] (See Figure 1) In addition, we discuss: (a) sample size and follow-up duration; and (b) ethical considerations.

Population, sampling strategy and follow-up interval

For peer review only - http://bmjopen.bmj.com/site/about/guidelines.xhtml

In Mesoamerica, CKDu typically affects young men on the Pacific Coast. This population is dying in their 40s, often younger, from end stage renal disease.[15, 30] The disease appears to occur at a later age in South Asia, with few cases occurring in men in their 20s.[7, 31] Nevertheless, one might expect preliminary changes in GFR to occur early in adulthood. In general, the study population should include participants who are old enough to experience an identifiable decline in kidney function, but not older age-groups (e.g., >60 year-old) where the prevalence of CKD is already high in many populations globally (e.g. up to 10%). Thus, inclusion criteria should be tailored to the local disease profile, but the default approach should be to recruit participants aged 18-40 years-old (though 18-30 might be more appropriate in Central America, and 18-50 may be more appropriate in areas such as South Asia where age of onset appears older). The rationale for including people ≥18-year-old was based on definition on adult life, and may be lowered, especially in populations where the working life starts years earlier. A population-census should be conducted to identify all potential participants in the appropriate age range and either the entire population recruited, or a random sample selected. In either case, response rates by age and sex, should be reported.

The focus of these studies is to conduct aetiological research in those without traditional CKD/risk-factors at baseline, thus, the sample size estimates (see below) are based on following a cohort in which those with evidence pre-existing CKD, diabetes or hypertension have been excluded.[25] Diabetes can be diagnosed by self-report, use of medication, or lab tests (fasting serum glucose: ≥7.0 mmol/l or HbA1C ≥48mmol/mol),[32,

For peer review only - http://bmjopen.bmj.com/site/about/guidelines.xhtml

33) and hypertension by self-report, use of medication or measurement (seated, average BP ≥140/90mmHg on second and third of three readings).[(34)] In addition to self-report of CKD, those with previously detected eGFR<60 mL/min/1.73m^2, proteinuria, (e.g. albumin/creatinine ratio, ACR, >300mg/g or dipstick 3+ or greater)[(35)] on testing at baseline should be excluded from the study. However, for practical, ethical or scientific reasons (for example, to gain insight into progression of established disease), investigators may wish to study an entire population (including those with pre-existing clinical diagnosis of, or newly identified, CKD, diabetes mellitus, and hypertension), but in that case, it is important to ensure that there are sufficient 'disease free' participants included at baseline to meet the sample size requirements (see table 1). Although the disease is generally more common in men, women with CKDu are of strong scientific interest in that they may suggest alternative risk factors, or help to rule out some that have been previously proposed. Hence recruitment should in general involve equal numbers of males and females, though women who are pregnant at recruitment are also excluded, since pregnancy-related changes in eGFR are challenging to interpret.

The baseline study visit will require the administration of the core-questionnaire, with additional context-specific additions, clinical measurements and biological samples. Subsequent to the baseline visit, follow-up visits should be conducted at least annually for a minimum follow-up of two-years to evaluate the study outcome and keep close contact with the participants and update their contact information. This will help minimize the loss to follow-up at each study point. Substantial seasonal variation in eGFR has been

For peer review only - http://bmjopen.bmj.com/site/about/guidelines.xhtml

reported in a number of settings (both CKDu related and unrelated).[26, 36-38] Therefore, the conduct of additional study visits at a 6-monthly interval (e.g. at beginning and end of summer season) might be useful in explaining within-person eGFR variation as well as providing important information for the wider population on the significance of kidney function testing at different time point in the year (perhaps for a subset of participants or a proportion of the follow-up period). (See table 2)

Questionnaires

The purpose of the baseline core-questionnaire is to obtain a minimum dataset to explore associations with decline in kidney function and make comparisons within and between persons. The baseline core-questionnaire (supplementary file 1) is based on the questionnaire used in the DEGREE protocol and has been used in DEGREE-related studies in a number of settings. The baseline core-questionnaire represents a minimum data set, and local research teams may decide to add data items of specific interest to the core dataset, particularly items of relevance to societal and occupational context. They also have the responsibility to translate, validate, and to make any local contextual changes. Training procedures for the field-staff should be documented.

Researchers will return to field (at least) annually for in-person follow-up visits. All participants have to respond a follow-up questionnaire (supplementary file 2) and update their contact information.

For peer review only - http://bmjopen.bmj.com/site/about/guidelines.xhtml

Clinical measurements

Blood pressure and heart rate should be measured on the right arm after 5 minutes rest in the sitting position using an automated sphygmomanometer, WHO validated for the clinical setting (example: Omron HEM-907XL sphygmomanometer) and the average of the second and third of three readings recorded. Subjects height and weight (in centimetres and kilograms) should be measured (without shoes) using a stadiometer and digital calibrated scales.

Biosamples

Fasting blood and urine samples will be collected at each study visit and stored in the field into coolers with icebox (4ºC) no more than 4 hours before processing.

Dipstick urinalysis should be performed by using electronic readers (urine chemistry analyser) where possible, or otherwise at least 10% of tests should be re-analysed by a second investigator. Parameters that should be reported are: urinary specific gravity, pH, protein, blood, leucocytes, nitrite, glucose, etc. Investigators with access to ACR measurements may wish to perform these assays (at least at baseline).

Samples for serum analysis should be centrifuged at 3500 rpm for 10 minutes within 4 hours of collection, and subsequently separated into at least four aliquots of 1-2 mL and stored at ≤-20ºC (ideally -80ºC). One aliquot should be used for contemporary sCr measurements e.g. by using the modified Jaffe assay (ideally also using standards

For peer review only - http://bmjopen.bmj.com/site/about/guidelines.xhtml

traceable to isotope dilution mass spectrometry [IDMS] reference material). At baseline and during each study visit a cross-checking of local lab quality control is highly recommended to ensure that sCr determinations are comparable as these lab results may guide referral to clinical care for participants during the follow-up period.

A further aliquot should be stored for batch measurement of sCr at the end of follow-up using a method traceable to an IDMS reference material (and potentially also cystatin C). The CO-DEGREE group suggest the storage of at least a further two 1-2mL aliquots of serum and a similar amount of urine in addition to those described above. Additional samples and analyses should be pursued depending on the priorities of the local research team. All samples for future analysis should be stored at ≤-20ºC (ideally -80ºC) in a local or international biobank. Such a biobank requires an uninterruptible power supply to protect the samples.

Investigators should assess and obtain consent from participants for future use of samples for further analyses both locally and internationally (e.g. through the DEGREE collaboration) as well as ensure that storage capacity is available.

Data management and reporting

Questionnaires and samples will be labelled using a unique bar-code to maintain participant confidentiality. Electronic data capture systems such as Open Data Kit [39] may be the most resource efficient method to capture questionnaire data but where hard-

For peer review only - http://bmjopen.bmj.com/site/about/guidelines.xhtml

copies are used double data-entry should be undertaken to minimise the transcription errors.

The CO-DEGREE protocols are openly available to interested research teams. Although primarily designed to be used in population-based studies similar approaches could also be used in an occupational or other selected cohorts.

Each centre will be 'owner' of their data and expected to publish the results of their study independently. However, where a study is registered as part of the DEGREE collaboration the coordinating centre will request a digital copy of anonymized individual-level data to allow the undertaking of international comparisons. In addition, a summary of local contextual information and a description of the population characteristics along with response rates will be requested. The importance of such information is emphasized.

Sample size and follow-up duration

The overall size of the cohort will be largely dependent on the proportion of the 'healthy' population which is expected to experience a 'substantial' decline in eGFR over time in the community as a result of CKDu. As discussed above, demonstrating that reduced renal function without diabetes, hypertension, or known kidney diseases is prevalent on a cross-sectional basis is a necessary first step before pursuing this work. If for example this study protocol was to be conducted in a general population sample in Europe or the

For peer review only

For peer review only - http://bmjopen.bmj.com/site/about/guidelines.xhtml

USA with similar exclusion criteria, then CKDu would be virtually non-existent, and there would also be very little or no decline of kidney function in the young adult population. In contrast, in our Nicaragua study of apparently healthy adults aged 18-30 years,[25, 26] there was a clearly distinct subgroup which experienced a marked decline in kidney function over a short time, whereas the eGFR in the other study participants was relatively stable. Given this distribution of such eGFR trajectories in the population we would expect any analysis of risk factors to be conducted using a prospective case-control approach.

Therefore, the sample size requirements to detect an association with an exposure at any given power will be determined by the following factors:

1. Proportion of the population that experience 'substantial' decline

   In turn the power to detect 'substantial' decline will depend on:

   a) The rate of eGFR decline in those affected
   b) The duration of follow-up
   c) The number of eGFR measures
2. Proportion of general population exposed to any exposure of interest
3. Effect size of any exposure
4. The study retention rate

Taking a simplistic approach, the duration of the study should be designed so that those affected have sustained a clinically important loss of kidney function, e.g. 20% of normal eGFR. Therefore, if CKDu in the study population is predicted, from a baseline of

For peer review only

For peer review only - http://bmjopen.bmj.com/site/about/guidelines.xhtml

≥60ml/min eGFR, to lead to a loss of eGFR of a magnitude of 5% each year (~7mL/min/1.73 m^2/year) the study duration should be 4 years. If alternatively, loss is predicted to be 10% each year study duration could be as short as 2 years. Additional eGFR measures, over and above the suggested annual frequency will reduce error associated with determining trajectory (and might be performed for the reasons discussed above) but either way a minimum follow-up of 2 years is recommended.

After basing the study duration on the expected rate of eGFR decline among those affected, the sample size can then be calculated on the basis of the expected frequency of ‘substantial’ decline amongst the population and the effect size of any proposed exposure that it is desirable to detect. A number of scenarios are outlined in Table 1. A further (e.g. 20%, depending on local circumstances) increase in target recruitment is advised to allow for loss to follow-up.

Finally, these initial sample sizes will need adjustment for exclusions based on estimates of the prevalence of previously unknown CKD (based on eGFR/albuminuria tests), diabetes, hypertension or other known causes of CKD at baseline (unless these data are already available from a previously conducted cross-sectional study). It is worth considering whether people who may have CKD (or CKD risk factors) will be aware of this, as this may affect the numbers of participants that will be retained for the analysis following testing. For example, if there is screening for kidney problems (as in some Central American Sugarcane mills or community-based screening in Sri Lanka), then

For peer review only - http://bmjopen.bmj.com/site/about/guidelines.xhtml

potential cohort participants may be aware of their kidney function status and can be excluded from the study sample prior to recruitment. For example, 5% of the target population in the community studied in Nicaragua reported pre-existing CKD. Nevertheless, there was an additional 10% who had undiagnosed impaired kidney function at baseline assessment based on their laboratory records, highlighting the importance of identifying an age-group where CKDu is not already highly prevalent so as to satisfy a key inclusion criterion (absence of CKD at baseline) when calculating sample sizes.

Ethics/regulatory issues and dissemination

Local research teams will ensure these studies are conducted in accordance with the Declaration of Helsinki Principles and be responsible for assuring that the work is approved by the local institutional review board (IRBs). Written informed consent will be obtained from all participants before taking part in the study. Information should be transparent in terms of using the data and biosamples stored for future research. Typically, a key aspect of the ethical review of any protocol is a discussion surrounding the provision of feedback and advice to participants when abnormal results become available. In most settings these processes should be developed in partnership with local communities. Furthermore, mechanisms will need to be established in collaboration with local health providers/healthcare systems to define pathways for participants needing referral for medical care. Findings from these studies should be disseminated widely by

For peer review only - http://bmjopen.bmj.com/site/about/guidelines.xhtml

publication in peer-reviewed journals and presentations/representations to relevant local stake holders.

Experience with the CO-DEGREE protocol in Nicaragua

The protocol presented here is, by necessity, generic. The approaches and challenges of implementing the protocol will vary widely in different populations and regions of the world. However, since we have already implemented this protocol in a study in Nicaragua,[25, 26] we will make some observations on the practicalities, and challenges, or implementing the protocol in this context.

The Nicaragua study involved community-based follow-up in Leon and Chinandega departments.[25] A number of strategies were used to maximise response and retention rates. As the workday starts very early in the morning and finishes late in the afternoon attempts were made to conduct data collection during economically less active (e.g. each side of the main sugar harvest) periods of the year, so as to still capture approximately 30% of participants who were employed at the time. Additionally, participants receive their kidney test results within a fortnight of the study visits and receive reimbursement of expenses and any lost income they have incurred to attend the study visit. Although study visits have been timetabled to occur outside of the harvest season, employees still express the concern that their employment opportunities might be affected by taking part in the study. In an attempt to mitigate against these types of consequences, the study team have corresponded with local employers explaining the content and extent of this

For peer review only - http://bmjopen.bmj.com/site/about/guidelines.xhtml

study in order to reduce any concerns about workers' participation. In addition, the study team takes particular precautions to maintain participant's confidentiality during the study and beyond.

Conducting a follow up study in a rural area remains a major challenge. Alongside the logistical challenges of reaching geographically isolated neighbourhoods along poor quality roads, a significant obstacle has been internal and external migration due to lack of employment source or social unrest. Rural communities have a tradition of working with seasonal crops and sugarcane workers often leave their communities at the end of each harvest season, to go abroad or to other regions within the country in search of temporary employment. At the end of each harvest, up to 30% of the study population had left their communities in search of alternative employment during the non-harvest period in our study. Despite these problems our team achieved attendance at 92% of all scheduled visits over two years.(25, 26) However the level of investment of time and resources should not be underestimated.

Finally, continuing community engagement and the maintenance of good relationships between researchers, community leaders, participants and communication with local health care system have been key. E.g., a reference flowchart for communication with local health posts/primary hospital or hospital for persons with health problems detected during the study.

For peer review only - http://bmjopen.bmj.com/site/about/guidelines.xhtml

Discussion

The CO-DEGREE protocol was developed in response to the highly prevalent form of CKD of unknown cause that is affecting Mesoamerica and other countries around the globe. To date, the existing epidemiological studies of CKDu have provided an incomplete understanding of the evolution of and risk factors for disease. This CO-DEGREE protocol aims to provide a framework to address this.

This CO-DEGREE protocol is designed to capture the entire at-risk population by aiming to recruit men and women, and those that work across a variety of different occupations. The main outcome measure of within-person loss of eGFR over time, which means it is should be possible to capture the earliest disease stages of disease, making associations with possible causal exposures (and exacerbating factors) less prone to reverse causation and recall bias.

We do not underestimate the challenges posed by the lack of language-validated and standardized exposure questionnaires in this area. The accompanying questionnaire represents a minimum and most studies will utilise an expanded dataset. Currently there is an absence of globally generalizable instruments to capture environmental and occupational exposures, however the DEGREE group is undertaking further work in this area. Additionally, short or long-term environmental measurements and/or novel biomarkers that capture exposure to heat, agrichemicals, and/or infection in either the

For peer review only

For peer review only - http://bmjopen.bmj.com/site/about/guidelines.xhtml

community or workplace are likely to be valuable additions to this type of study but are beyond the scope of this basic protocol.

Finally, it should be emphasized that this protocol is not suitable for studying the progression of CKD in general, due to the specific constraints introduced by excluding those with hypertension, diabetes and CKD as well as other known causes of CKD (i.e. those with proteinuria and/or with reduced eGFR) at baseline. Indeed, in settings where there is not a high prevalence of CKDu, a cohort comprised of people without traditional risk factors for CKD or with CKD would be unlikely to identify any detectable kidney function loss over time in the young-adult population. For studies outside the CKDu arena, investigators are advised to use alternative methodologies using established protocols, for example, the CRIC study.[40]

In conclusion, we have designed a CO-DEGREE protocol that can be used in the different settings around the globe to investigate the evolution of CKDu and associated risk factors for decline in kidney function. These studies should provide important information on the early decline in kidney function across different affected areas as well as key insight into the cause(s) of disease.

For peer review only - http://bmjopen.bmj.com/site/about/guidelines.xhtml

Reference

1. Wegman D, Crowe J, Hogstedt C, Jakobsson K, Wesseling C, editors. Mesoamerican nephropathy: report from the second international research workshop on MeN. Heredia, C.R: SALTRA/IRET-UNA; 2016. Report No.: ISBN 978-9968-924-33-7.
2. Torres C, Aragon A, Gonzalez M, Lopez I, Jakobsson K, Elinder CG, et al. Decreased kidney function of unknown cause in Nicaragua: a community-based survey. Am J Kidney Dis. 2010;55(3):485-96.
3. O'Donnell JK, Tobey M, Weiner DE, Stevens LA, Johnson S, Stringham P, et al. Prevalence of and risk factors for chronic kidney disease in rural Nicaragua. Nephrol Dial Transplant. 2011;26(9):2798-805.
4. Orantes CM, Herrera R, Almaguer M, Brizuela EG, Hernandez CE, Bayarre H, et al. Chronic kidney disease and associated risk factors in the Bajo Lempa region of El Salvador: Nefrolempa study, 2009. MEDICC Review. 2011;13(4):14-22.
5. Orantes CM, Herrera R, Almaguer M, Brizuela EG, Nunez L, Alvarado NP, et al. Epidemiology of chronic kidney disease in adults of Salvadoran agricultural communities. MEDICC Review. 2014;16(2):23-30.
6. Jayasekara JM, Dissanayake DM, Adhikari SB, Bandara P. Geographical distribution of chronic kidney disease of unknown origin in North Central Region of Sri Lanka. Ceylon Med J. 2013;58(1):6-10.
7. Jayatilake N, Mendis S, Maheepala P, Mehta FR, Team CKNRP. Chronic kidney disease of uncertain aetiology: prevalence and causative factors in a developing country. BMC Nephrol. 2013;14:180.
8. Ganguli A. Uddanam Nephropathy/Regional Nephropathy in India: Preliminary Findings and a Plea for Further Research. Am J Kidney Dis. 2016;68(3):344-8.
9. Jayasumana C, Orantes C, Herrera R, Almaguer M, Lopez L, Silva LC, et al. Chronic interstitial nephritis in agricultural communities: a worldwide epidemic with social, occupational and environmental determinants. Nephrol Dial Transplant. 2017;32(2):234-41.
10. Peraza S, Wesseling C, Aragon A, Leiva R, Garcia-Trabanino RA, Torres C, et al. Decreased kidney function among agricultural workers in El Salvador. Am J Kidney Dis. 2012;59(4):531-40.
11. García-Trabanino R, Cerdas M, Madero M, Jakobsson K, Barnoya J, Crowe J, et al. Nefropatía mesoamericana: revisión breve basada en el segundo taller del Consorcio para el estudio de la Epidemia de Nefropatía en Centroamérica y México (CENCAM). Nefrología Latinoamericana. 2017;14(1):39-45.
12. Lozier M, Turcios-Ruiz RM, Noonan G, Ordunez P. Chronic kidney disease of nontraditional etiology in Central America: a provisional epidemiologic case definition for surveillance and epidemiologic studies. Rev Panam Salud Publica. 2016;40(5):294-300.
13. Rajapakse S, Shivanthan MC, Selvarajah M. Chronic kidney disease of unknown etiology in Sri Lanka. Int J Occup Environ Health. 2016;22(3):259-64.
14. WHO. Workshop report: Designing a step-wise approach to estimate the burden and to understand the etiology of CKDu in Sri Lanka. Sri Lanka: WHO; 2016, 24-25th October.

For peer review only - http://bmjopen.bmj.com/site/about/guidelines.xhtml

15. Ordunez P, Nieto FJ, Martinez R, Soliz P, Giraldo GP, Mott SA, et al. Chronic kidney disease mortality trends in selected Central America countries, 1997-2013: clues to an epidemic of chronic interstitial nephritis of agricultural communities. J Epidemiol Community Health. 2018;72(4):280-6.
16. Ordunez P, Saenz C, Martinez R, Chapman E, Reveiz L, Becerra F. The epidemic of chronic kidney disease in Central America. Lancet Glob Health. 2014;2(8):e440-1.
17. Ekiti ME, Zambo JB, Assah FK, Agbor VN, Kekay K, Ashuntantang G. Chronic kidney disease in sugarcane workers in Cameroon: a cross-sectional study. BMC Nephrol. 2018;19(1):10.
18. Ministry of Health Nutrition and Indigenous Medicine - Medical Statistics Unit. Annual health bulletin of Sri Lanka 2015. Sri Lanka: Ministry of Health, Nutrition and Indigenous Medicine; 2017 [cited 2017 June 20]. Available from: http://www.health.gov.lk/moh_final/english/public/elfinder/files/publications/AHB/2017/AHB%202015.pdf.
19. Nanayakkara S KT, Rajapurkar MM, John GT, Kirpalani AL. . What do we know about chronic kidney disease in India: first report of the Indian CKD registry. . BMC Nephrol 2012;13(10).
20. Correa-Rotter R, Wesseling C, Johnson RJ. CKD of unknown origin in Central America: the case for a Mesoamerican nephropathy. Am J Kidney Dis. 2014;63(3):506-20.
21. Wesseling C, Crowe J, Hogstedt C, Jakobsson K, Lucas R, Wegman DH. The epidemic of chronic kidney disease of unknown etiology in Mesoamerica: a call for interdisciplinary research and action. Am J Public Health. 2013;103(11):1927-30.
22. Wesseling C, Crowe J, Hogstedt C, Jakobsson K, Lucas R, Wegman D, et al. First international research workshop on mesoamerican nephropathy (MeN). Heredia, C.R.: SALTRA/IRET-UNA; 2013. Report No.: ISBN 978-9968-924-06-1.
23. Caplin B, Jakobsson K, Glaser J, Nitsch D, Jha V, Singh A, et al. International Collaboration for the Epidemiology of eGFR in Low and Middle Income Populations - Rationale and core protocol for the Disadvantaged Populations eGFR Epidemiology Study (DEGREE). BMC Nephrol. 2017;18(1):1.
24. Gonzalez-Quiroz M, Pearce N, Caplin B, Nitsch D. What do epidemiological studies tell us about chronic kidney disease of undetermined cause in Meso-America? A systematic review and meta-analysis. Clin Kidney J. 2018;11(4):496-506.
25. Gonzalez-Quiroz M, Camacho A, Faber D, Aragon A, Wesseling C, Glaser J, et al. Rationale, description and baseline findings of a community-based prospective cohort study of kidney function amongst the young rural population of Northwest Nicaragua. BMC Nephrol. 2017;18(1):16.
26. Gonzalez-Quiroz M, Smpokou ET, Silverwood RJ, Camacho A, Faber D, Garcia BR, et al. Decline in Kidney Function among Apparently Healthy Young Adults at Risk of Mesoamerican Nephropathy. J Am Soc Nephrol. 2018;29(8):2200-12.
27. Caplin Ben, González-Quiroz Marvin, Pearce Neil. Gaining insights into the evolution of CKDnt from community-based follow up studies. In: SALTRA, editor. Second International Workshop on Mesoamerican Nephropathy; San José. Costa Rica: SALTRA; 2015.

For peer review only - http://bmjopen.bmj.com/site/about/guidelines.xhtml

28. Padala S, Tighiouart H, Inker LA, Contreras G, Beck GJ, Lewis J, et al. Accuracy of a GFR estimating equation over time in people with a wide range of kidney function. Am J Kidney Dis. 2012;60(2):217-24.
29. Inker LA, Schmid CH, Tighiouart H, Eckfeldt JH, Feldman HI, Greene T, et al. Estimating glomerular filtration rate from serum creatinine and cystatin C. N Engl J Med. 2012;367(1):20-9.
30. Garcia-Trabanino R, Trujillo Z, Colorado AV, Magana Mercado S, Henriquez CA, En nombre de la Asociacion de Nefrologia e Hipertension Arterial de El S. Prevalence of patients receiving renal replacement therapy in El Salvador in 2014. Nefrologia. 2016;36(6):631-6.
31. Jha V, Garcia-Garcia G, Iseki K, Li Z, Naicker S, Plattner B, et al. Chronic kidney disease: global dimension and perspectives. Lancet. 2013;382(9888):260-72.
32. American Diabetes A. 2. Classification and Diagnosis of Diabetes: Standards of Medical Care in Diabetes-2018. Diabetes Care. 2018;41(Suppl 1):S13-S27.
33. Association American D. Updates to the Standards of Medical Care in Diabetes-2018. Diabetes Care. 2018;41(9):2045-7.
34. Chobanian AV, Bakris GL, Black HR, Cushman WC, Green LA, Izzo JL, Jr., et al. Seventh report of the Joint National Committee on Prevention, Detection, Evaluation, and Treatment of High Blood Pressure. Hypertension. 2003;42(6):1206-52.
35. KDIGO. KDIGO 2012 Clinical Practice Guideline for the Evaluation and Management of Chronic Kidney Disease. Kidney Int Supps. 2013;3(1):1-150.
36. Wesseling C, Aragon A, Gonzalez M, Weiss I, Glaser J, Bobadilla NA, et al. Kidney function in sugarcane cutters in Nicaragua--A longitudinal study of workers at risk of Mesoamerican nephropathy. Environ Res. 2016;147:125-32.
37. Wesseling C, Aragon A, Gonzalez M, Weiss I, Glaser J, Rivard CJ, et al. Heat stress, hydration and uric acid: a cross-sectional study in workers of three occupations in a hotspot of Mesoamerican nephropathy in Nicaragua. BMJ Open. 2016;6(12):e011034.
38. Laws RL, Brooks DR, Amador JJ, Weiner DE, Kaufman JS, Ramirez-Rubio O, et al. Changes in kidney function among Nicaraguan sugarcane workers. Int J Occup Environ Health. 2015;21(3):241-50.
39. Open Data KIT. Longitudinal Clinic Study App: GitHub, Inc; 2018 [cited 2018 July 28]. Available from: https://opendatakit.org/use/2_0_tools/odk-application-designer-2-0-rev126/.
40. Feldman HI, Appel LJ, Chertow GM, Cifelli D, Cizman B, Daugirdas J, et al. The Chronic Renal Insufficiency Cohort (CRIC) Study: Design and Methods. J Am Soc Nephrol. 2003;14(7 Suppl 2):S148-53.

For peer review only

For peer review only - http://bmjopen.bmj.com/site/about/guidelines.xhtml

Authors´contributions

All authors contributed to the drafting of the manuscript as well as the concept and design of the protocol.

Funding statement

This work was supported by grants from the UK Colt Foundation and the UK Medical Research Council (MR/P02386X/1).

Competing interests statement

The authors declare they have not competing interests

For peer review only

For peer review only - http://bmjopen.bmj.com/site/about/guidelines.xhtml

DEGREE Study Steering Committee

Neil Pearce (UK) (Chair)
Ben Caplin (UK) (Co-chair)
Jason Glaser (USA)
Ricardo Correa-Rotter (Mexico)
Kristina Jakobsson (Sweden)
Ajay Singh (USA/India)

Antonio Bernabe-Ortiz (Peru)
Emmanuel Burdmann (Brazil)
Marvin Gonzalez (Nicaragua)
Vivekanand Jha (India)
Rick Johnson (USA)
Phabdheep Kaur (India)
Pronpimolk Kongtip (Thailand)
Hans Kromhout (Netherlands)
Adeera Levin (Canada)
Magdalena Madero Rovalo (Mexico)
Dorothea Nitsch (UK)
Moffat Nyirenda (Ugand/Malawi)
Cristina O'Callaghan-Gordo (Spain)
Pablo Perel (UK/Argentina)
Dorairaj Prabhkaran (India)
Narayan Prasad (India)
Giuseppe Remuzzi (Italy)
Rajiv Saran (USA)
Liam Smeeth (UK)
Vidhya Venugopal (India)

Observers
Nalika Gunawardenan (Sri Lanka)

For peer review only - http://bmjopen.bmj.com/site/about/guidelines.xhtml

List of figures and tables

Figures:

Figure 1: Flow chart and study procedures of CO-DEGREE protocol

Tables:

Table 1: Sample Size Calculations

Table 2. Details and procedures of the baseline study visit and subsequence follow-up.

Supplementary material

CO-DEGREE baseline questionnaire

CO-DEGREE follow-up questionnaire

For peer review only - http://bmjopen.bmj.com/site/about/guidelines.xhtml

Table 1: Sample Size Calculations

Parameters	Scenario 1	Scenario 2	Scenario 3	Scenario 4	Scenario 5	Scenario 6	Scenario 7	Scenario 8
**Population frequency of eGFR decline**	0.04	0.06	0.08	0.10	0.04	0.06	0.08	0.10
**Proportion population exposed**	0.5							
**Odds ratio associated with exposure**	2				3			
**P (outcome|unexposed)**	0.027	0.04	0.053	0.066	0.02	0.03	0.04	0.05
**P (outcome|exposed)**	0.054	0.08	0.106	0.132	0.06	0.09	0.12	0.15
**Group size**	993	686	405	436	463	317	243	200
**Sample size**	**1986**	**1372**	**810**	**872**	**926**	**634**	**486**	**400**

Abbreviations, eGFR, estimated glomerular filtration rate; P: probability. Assumes $1-\beta=0.80$; $=0.05$; Calculations based on equal proportion of the population exposed/unexposed for simplicity. No adjustments made for loss to follow-up or multiple testing.

For peer review only

Table 2. Details and procedures of the baseline study visit and subsequence follow-up.

Items	***Baseline visit (0 month)***	***Follow-up period (variable)***				
		12 months	*24 months*	*36 months*	*48 months*	*At completion*
Community census	X	-	-	-	-	-
Participants enrolment	X	-	-	-	-	-
Informed consent	X					
Update personnel contact information	X	X	X	X	X	
Anthropometric measurements	X	X	X	X	X	
Biological samples	X	X	X	X	X	
Baseline core-questionnaire	X	-	-	-	-	
Follow-up questionnaire		X	X	X	X	
Local serum creatinine measurement	X	X	X	X	X	
Results feedback	X	X	X	X	X	
Biobank	X	X	X	X	X	
Batch testing of serum creatinine						X

For peer review only - http://bmjopen.bmj.com/site/about/guidelines.xhtml

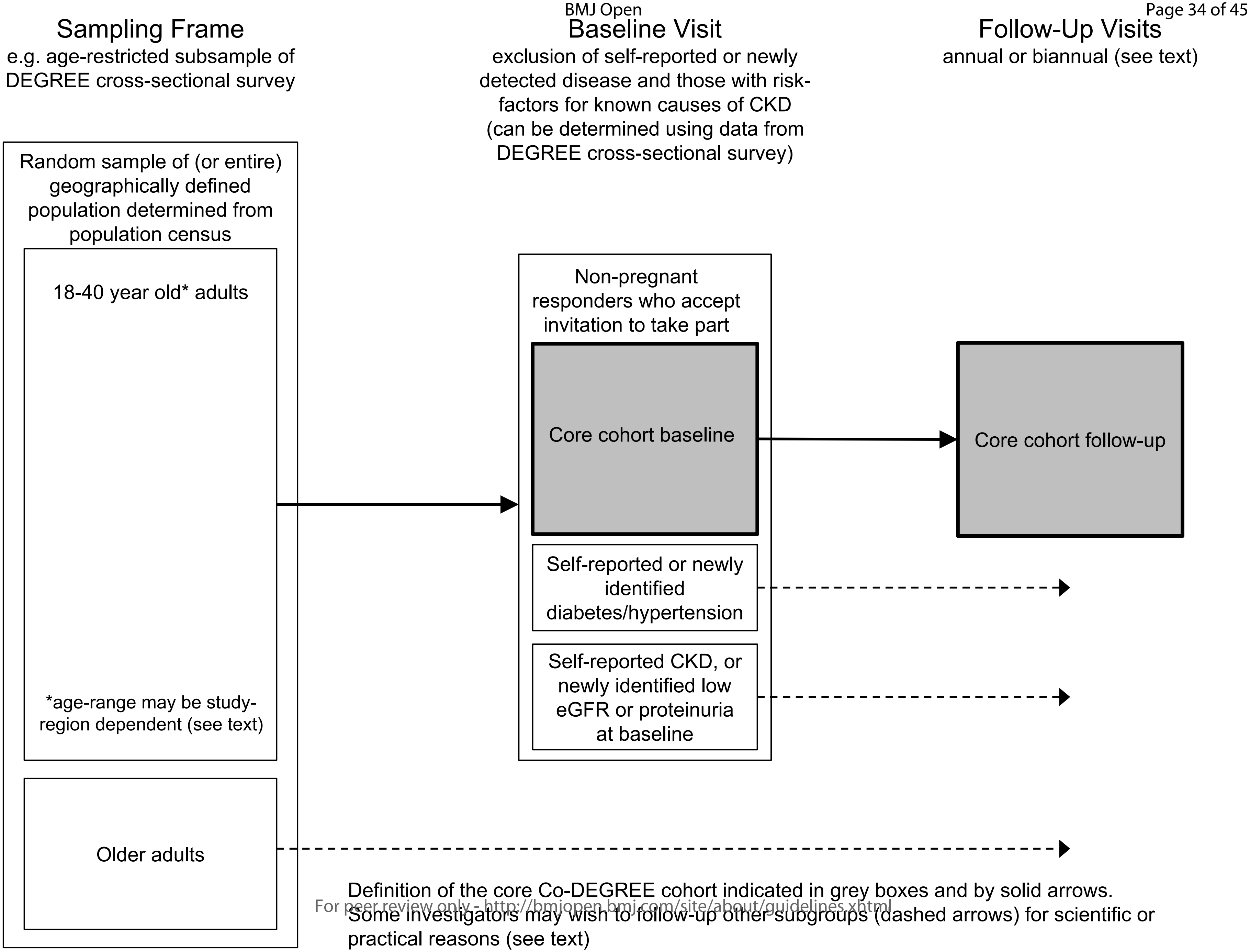

Definition of the core Co-DEGREE cohort indicated in grey boxes and by solid arrows. Some investigators may wish to follow-up other subgroups (dashed arrows) for scientific or practical reasons (see text)

For peer review only - http://bmjopen.bmj.com/site/about/guidelines.xhtml

CO-DEGREE: baseline questionnaire

PASTE THE ID LEVEL HERE

Participant Identification Number └─┴─┴─┘ └─┴─┴─┘ └─┴─┴─┘

CO-DEGREE Basic Core Questionnaire

	Response	Code
Study site ID	└─┴─┴─┘	I1
Interviewer ID	└─┴─┴─┘	I2
Study visit number	└─┴─┴─┘	I3
Date of completion of the instrument	└─┴─┘ └─┴─┘ └─┴─┴─┴─┘<br>dd mm year	I4

Consent, Interview Language and Name	Response	Code
Consent has been read and obtained	Yes 1<br>No 2 If NO, END	I5
Interview Language *[Insert Language]*	English 1<br>*[Add others]* 2<br>*[Add others]* 3<br>*[Add others]* 4	I6
Time of interview<br>(24 hour clock)	└─┴─┘ : └─┴─┘<br>hrs mins	I7
Family Surname		I8
First Name		I9
Address:		
Additional Information that may be helpful		
Contact phone number where possible		I10

CO-DEGREE: baseline questionnaire

PASTE THE ID LEVEL HERE

Participant Identification Number |___|___|___| |___|___|___| |___|___|___|

CO-DEGREE basic core clinical and anthropometry measurements

Question		Response	Code
Ambient Temperature<br>(at time of examination measured in shade)		|___|___|___| °C	Temp
**Blood Pressure**			
Question		Response	Code
Interviewer ID		|___|___|___|	M1
Device ID for blood pressure		|___|___|___|	M2
Cuff size used	Small<br>Medium<br>Large	1<br>2<br>3	M3
Reading 1	Systolic (mmHg)	|___|___|___|	M4a
	Diastolic (mmHg)	|___|___|___|	M4b
	Heart rate	|___|___|___|	M4c
Reading 2	Systolic (mmHg)	|___|___|___|	M5a
	Diastolic (mmHg)	|___|___|___|	M5b
	Heart rate	|___|___|___|	M5c
Reading 3	Systolic (mmHg)	|___|___|___|	M6a
	Diastolic (mmHg)	|___|___|___|	M6b
	Heart rate	|___|___|___|	M6c
During the past two weeks, have you been treated for raised blood pressure with drugs (medication) prescribed by a doctor or other health worker?	Yes<br>No	1<br>2	M7
**Height, and Weight**			
For women: Are you pregnant?	Yes<br>No	1 if yes should be excluded<br>2	M8
Have you eaten yet today?	Yes<br>No	1<br>2	M9
Interviewer ID		|___|___|___|	M10
Height	in Centimetres (cm)	|___|___|___|	M11
Weight<br>*If too large for scale 666.6*	in Kilograms (kg)	|___|___|___|	M12

For peer review only - http://bmjopen.bmj.com/site/about/guidelines.xhtml

CO-DEGREE: baseline questionnaire

PASTE THE ID LEVEL HERE

Participant Identification Number |__|__|__| |__|__|__| |__|__|__|

CO-DEGREE basic core questionnaire (adapted from DEGREE PROTOCOL)

Question	Response	Code
Sex (*Record Male / Female as observed*)	Male 1<br>Female 2	C1
What is your date of birth?<br>*Don't Know 77 77 7777*	dd mm year	C2
How old are you?	Years |__|__|__|	C3
In total, how many years have you spent at school and in full-time study (excluding pre-school)?	Years |__|__|__|	C4
What is your *[insert relevant ethnic group / racial group / cultural subgroup / others]* background?	*[Locally defined]* 1<br>*[Locally defined]* 2<br>*[Locally defined]* 3<br>Refused 88	C6
Which of the following best describes your main work status over the past 12 months?	Government employee 1<br>Non-government employee 2<br>Self-employed 3<br>Non-paid 4<br>Student 5<br>Homemaker 6<br>Retired 7<br>Unemployed (able to work) 8<br>Unemployed (unable to work) 9<br>Unpaid domestic 10<br>Refused 88	C8
If you are working what is your main occupation [FREE TEXT]:		OCCTXT
What task do you perform? [FREE TEXT]		TASKTXT
How many years have you been working in your current job?	Years |__|__|__|	C9
How many hours do you work daily?	Hours |__|__|__|	C10
Where do you work mostly?	Indoors 1<br>Outdoors 2<br>Both 3	C11
Do you take work breaks in shade?	Yes 1<br>No 2	C12
Do you work in a very hot working environment?	Seldom or never 1<br>Few times 2<br>Regularly 3<br>Frequently 4<br>Always or almost always 5	C13
How much physical effort did you do at work?	Slight effort 1<br>Moderate effort 2<br>Hard effort 3<br>Very hard effort 4	C14
Do you have experience of migrant work?<br>[Defined as staying far from home for seasonal work]	Yes *1*<br>No 2	MIGR
Can you give an estimate of the monthly household income if I read some options to you? Is it<br>*[INSERT QUINTILE VALUES IN LOCAL CURRENCY]*<br><br>*(READ OPTIONS)*	≤ Quintile (Q) 1 1<br>More than Q 1, ≤ Q 2 2<br>More than Q 2, ≤ Q 3 3<br>More than Q 3, ≤ Q 4 4<br>More than Q 4 5<br>Don't Know 77<br>Refused 88	C15

For peer review only - http://bmjopen.bmj.com/site/about/guidelines.xhtml

CO-DEGREE: baseline questionnaire

PASTE THE ID LEVEL HERE

Participant Identification Number |_|_|_| |_|_|_| |_|_|_|

During the past 12 months, how frequently have you had at least one standard alcoholic drink?<br>*(READ RESPONSES, USE SHOWCARD)*	Daily 1<br>5-6 days per week 2<br>3-4 days per week 3<br>1-2 days per week 4<br>1-3 days per month 5<br>Less than once a month 6<br>Not at all 7<br>Refused 88	A4
Do you currently smoke any tobacco products, such as cigarettes, cigars or pipes? *(USE SHOWCARD)*	Yes 1<br>No 2	*T1*
In a typical week, on how many days do you eat MEAT *(USE SHOWCARD)*	Number of days |_|_| Don't Know 77	*D1*
Does your work involve vigorous-intensity activity that causes large increases in breathing or heart rate like *[carrying or lifting heavy loads, digging or construction work]* for at least 10 minutes continuously? *(OR USE SHOWCARD)*	Yes *1*<br>No 2	*P1*
Have you ever been told by a doctor or other health worker that you have raised blood pressure or hypertension?	Yes 1<br>No 2	H2a
Have you ever been told by a doctor or other health worker that you have raised blood sugar or diabetes?	Yes 1<br>No 2	H7a
Have you used agrichemicals?	Yes 1<br>No 2	L1
Did you mix, apply or both?	Mix 1<br>Apply 2<br>Both 3	L2
Have you been diagnosed with?	Dengue 1<br>Chikungunya 2<br>Zika 3<br>Malaria 4	L3
**Renal Protocol**		
Has a doctor diagnosed you with kidney disease?	No 1 go to question KI3<br>Yes 2 go to question KI2	KI1
Have you been told you have one of these kidney disease?	Glomerulonephritis 1<br>Congenital abnormality of the 2<br>kidneys 3<br>Polycystic kidney disease 4<br>Diabetic kidney disease 5<br>[locally defined] 6<br>[locally defined] 7<br>[locally defined] 8	KI2
Have you been told you have ever been told you have one of these diseases?	Tuberculosis 1<br>HIV 2<br>Hepatitis B 3<br>Hepatitis C 4<br>Schistosomiasis 5<br>Leptospirosis 6<br>[locally defined] 7<br>[locally defined] 8	KI3
Do you take herbal or traditional remedies?	No 1<br>Yes 2	KI4
Do you take regular prescribed medications?	No 1 go to question KI 9<br>Yes 2 2 go to questions below	KI5
Do you take medication for diabetes?	No 1<br>Yes 2	KI6
Do you take medication against HIV or hepatitis?	No 1<br>Yes 2	KI7
Do you take medication for tuberculosis?	No 1<br>Yes 2	KI8
Have you used painkillers most days for more than several months?<br>[Use Showcard with locally available medications]?	No 1<br>Yes 2	KI9

For peer review only - http://bmjopen.bmj.com/site/about/guidelines.xhtml

CO-DEGREE: baseline questionnaire

PASTE THE ID LEVEL HERE

Participant Identification Number |__|__|__| |__|__|__| |__|__|__|

DEGREE study core lab measurements

Question	Response	Code
Blood sampling Investigator ID	|__|__|__|	B2
Time of day blood specimen taken (24 hour clock)	Hours : minutes |__|__| : |__|__| hrs mins	B4
Creatinine measurement Technician ID	|__|__|__|	CR1
Creatinine measurement Device ID	|__|__|__|	CR2
Serum Creatinine	*to first decimal place if in mg/dL* |__|__|__|	CR3
Serum Creatinine Units	mg/dL 1<br>µMol/L 2	CR4
Urine sampling Investigator ID	|__|__|__|	UR1
Urinalysis Device ID	|__|__|__|	UR2
Urine Glucose	Negative 1<br>100mg/dL 2<br>250mg/dL 3<br>500mg/dL 4<br>1000mg/dL 5<br>>2000mg/dL 6	UR3
Urine Specific Gravity	1.000 1<br>1.005 2<br>1.010 3<br>1.015 4<br>1.020 5<br>1.025 6<br>1.030 7	UR4
Urinalysis Blood	Negative 1<br>Non-haemolysed trace 2<br>Non-haemolysed moderate 3<br>Haemolysed trace 4<br>Small (+) 5<br>Moderate (++) 6<br>Large (+++) 7	UR5
Urine pH	5.0 1<br>6.0 2<br>6.5 3<br>7.0 4<br>7.5 5<br>8.0 6<br>8.5 7	UR6
Urinalysis Protein	Negative 1<br>Trace 2<br>30mg/dL (+) 3<br>100mg/dL (++) 4<br>300mg/dL (+++) 5<br>>2000mg/dL 6	UR7
Urinalysis Nitrite	Negative 1<br>Positive 2	UR8
Urinalysis Leucocytes	Negative 1<br>Trace 2<br>Small (+) 3<br>Moderate (++) 4<br>Large (+++) 5	UR9

For peer review only

CO-DEGREE: follow-up questionnaire

PASTE THE ID LEVEL HERE

Participant Identification Number |_|_|_| |_|_|_| |_|_|_|

CO-DEGREE Basic Core Questionnaire

	Response	Code
Study site ID	|_|_|_|	I1
Interviewer ID	|_|_|_|	I2
Study visit number	|_|_|_|	I3
Date of completion of the instrument	|_|_| |_|_| |_|_|_|_| dd mm year	I4

Interview Language and Name	Response	Code
Interview Language *[Insert Language]*	English 1<br>*[Add others]* 2<br>*[Add others]* 3<br>*[Add others]* 4	I6
Time of interview (24 hour clock)	|_|_| : |_|_| hrs mins	I7
Family Surname		I8
First Name		I9
Address:		
Additional Information that may be helpful		
Contact phone number where possible		I10

For peer review only - http://bmjopen.bmj.com/site/about/guidelines.xhtml

CO-DEGREE: follow-up questionnaire

PASTE THE ID LEVEL HERE

Participant Identification Number |__|__|__| |__|__|__| |__|__|__|

CO-DEGREE basic core clinical and anthropometry measurements

Question	Response	Code
Ambient Temperature (at time of examination measured in shade)	|__|__|__| °C	Temp
**Blood Pressure**		
Question	Response	Code
Interviewer ID	|__|__|__|	M1
Device ID for blood pressure	|__|__|__|	M2
Cuff size used	Small 1<br>Medium 2<br>Large 3	M3
Reading 1	Systolic (mmHg) |__|__|__|	M4a
	Diastolic (mmHg) |__|__|__|	M4b
	Heart rate |__|__|__|	M4c
Reading 2	Systolic (mmHg) |__|__|__|	M5a
	Diastolic (mmHg) |__|__|__|	M5b
	Heart rate |__|__|__|	M5c
Reading 3	Systolic (mmHg) |__|__|__|	M6a
	Diastolic (mmHg) |__|__|__|	M6b
	Heart rate |__|__|__|	M6c
During the past two weeks, have you been treated for raised blood pressure with drugs (medication) prescribed by a doctor or other health worker?	Yes 1<br>No 2	M7
**Height, and Weight**		
For women: Are you pregnant?	Yes 1<br>No 2	M8
Have you eaten yet today?	Yes 1<br>No 2	M9
Interviewer ID	|__|__|__|	M10
Height	in Centimetres (cm) |__|__|__|	M11
Weight<br>*If too large for scale 666.6*	in Kilograms (kg) |__|__|__|	M12

For peer review only - http://bmjopen.bmj.com/site/about/guidelines.xhtml

CO-DEGREE: follow-up questionnaire

PASTE THE ID LEVEL HERE

Participant Identification Number |__|__|__|__| |__|__|__|__| |__|__|__|__|

CO-DEGREE basic core questionnaire (adapted from DEGREE PROTOCOL)

Question	Response	Code
Sex (*Record Male / Female as observed*)	Male 1<br>Female 2	C1
How old are you?	Years |__|__|__|	C3
Which of the following best describes your main work status over the past 12 months?	Government employee 1<br>Non-government employee 2<br>Self-employed 3<br>Non-paid 4<br>Student 5<br>Homemaker 6<br>Retired 7<br>Unemployed (able to work) 8<br>Unemployed (unable to work) 9<br>Unpaid domestic 10<br>Refused 88	C8
If you are working what is your main occupation [FREE TEXT]:		OCCTXT
What task do you perform? [FREE TEXT]		TASKTXT
How many years have you been working in your current job?	Years |__|__|__|	C9
How many hours do you work daily?	Hours |__|__|__|	C10
Where do you work mostly?	Indoors 1<br>Outdoors 2<br>Both 3	C9
Do you take work breaks in shade?	Yes 1<br>No 2	C10
Do you work in a very hot working environment?	Seldom or never 1<br>Few times 2<br>Regularly 3<br>Frequently 4<br>Always or almost always 5	C11
How much physical effort did you do at work?	Slight effort 1<br>Moderate effort 2<br>Hard effort 3<br>Very hard effort 4	C12
Do you have experience of migrant work?<br>[Defined as staying far from home for seasonal work]	Yes *1*<br>No 2	MIGR
Can you give an estimate of the monthly household income if I read some options to you? Is it<br>*[INSERT QUINTILE VALUES IN LOCAL CURRENCY]*<br>*(READ OPTIONS)*	≤ Quintile (Q) 1 1<br>More than Q 1, ≤ Q 2 2<br>More than Q 2, ≤ Q 3 3<br>More than Q 3, ≤ Q 4 4<br>More than Q 4 5<br>Don't Know 77<br>Refused 88	C13
During the past 12 months, how frequently have you had at least one standard alcoholic drink?<br>*(READ RESPONSES, USE SHOWCARD)*	Daily 1<br>5-6 days per week 2<br>3-4 days per week 3<br>1-2 days per week 4<br>1-3 days per month 5<br>Less than once a month 6<br>Not at all 7<br>Refused 88	A4
Do you currently smoke any tobacco products, such as cigarettes, cigars or pipes? *(USE SHOWCARD)*	Yes 1<br>No 2	*T1*

For peer review only - http://bmjopen.bmj.com/site/about/guidelines.xhtml

CO-DEGREE: follow-up questionnaire

PASTE THE ID LEVEL HERE

Participant Identification Number |__|__|__|__| |__|__|__|__| |__|__|__|__|

In a typical week, on how many days do you eat MEAT *(USE SHOWCARD)*	Number of days<br>Don't Know 77 |__|__|	*D1*
Does your work involve vigorous-intensity activity that causes large increases in breathing or heart rate like *[carrying or lifting heavy loads, digging or construction work]* for at least 10 minutes continuously? *(OR USE SHOWCARD)*	Yes *1*<br>No 2	*P1*
Have you ever been told by a doctor or other health worker that you have raised blood pressure or hypertension?	Yes 1<br>No 2	H2a
Have you ever been told by a doctor or other health worker that you have raised blood sugar or diabetes?	Yes 1<br>No 2	H7a
Have you used agrichemicals?	Yes 1<br>No 2	L1
Did you mix, apply or both?	Mix 1<br>Apply 2<br>Both 3	L2
Have you been diagnosed with?	Dengue 1<br>Chikungunya 2<br>Zika 3<br>Malaria 4	L3
**Renal Protocol**		
Has a doctor diagnosed you with kidney disease?	No 1 go to question KI3<br>Yes 2 go to question KI2	KI1
Have you been told you have one of these kidney disease?	Glomerulonephritis 1<br>Congenital abnormality of the 2<br>kidneys 3<br>Polycystic kidney disease 4<br>Diabetic kidney disease 5<br>[locally defined] 6<br>[locally defined] 7<br>[locally defined] 8	KI2
Have you been told you have ever been told you have one of these diseases?	Tuberculosis 1<br>HIV 2<br>Hepatitis B 3<br>Hepatitis C 4<br>Schistosomiasis 5<br>Leptospirosis 6<br>[locally defined] 7<br>[locally defined] 8	KI3
Do you take herbal or traditional remedies?	No 1<br>Yes 2	KI4
Do you take regular prescribed medications?	No 1 go to question KI 9<br>Yes 2 2 go to questions below	KI5
Do you take medication for diabetes?	No 1<br>Yes 2	KI6
Do you take medication against HIV or hepatitis?	No 1<br>Yes 2	KI7
Do you take medication for tuberculosis?	No 1<br>Yes 2	KI8
Have you used painkillers most days for more than several months?<br>[Use Showcard with locally available medications]?	No 1<br>Yes 2	KI9

For peer review only - http://bmjopen.bmj.com/site/about/guidelines.xhtml

CO-DEGREE: follow-up questionnaire

PASTE THE ID LEVEL HERE

Participant Identification Number |_|_|_| |_|_|_| |_|_|_|

DEGREE study core lab measurements

For peer review only

For peer review only - http://bmjopen.bmj.com/site/about/guidelines.xhtml

CO-DEGREE: follow-up questionnaire

PASTE THE ID LEVEL HERE

Participant Identification Number |__|__|__| |__|__|__| |__|__|__|

Question	Response	Code
Blood sampling Investigator ID	|__|__|__|	B2
Time of day blood specimen taken (24 hour clock)	Hours : minutes |__|__| : |__|__| hrs mins	B4
Creatinine measurement Technician ID	|__|__|__|	CR1
Creatinine measurement Device ID	|__|__|__|	CR2
Serum Creatinine	*to first decimal place if in mg/dL* |__|__|__|	CR3
Serum Creatinine Units	mg/dL 1<br>µMol/L 2	CR4
Urine sampling Investigator ID	|__|__|__|	UR1
Urinalysis Device ID	|__|__|__|	UR2
Urine Glucose	Negative 1<br>100mg/dL 2<br>250mg/dL 3<br>500mg/dL 4<br>1000mg/dL 5<br>>2000mg/dL 6	UR3
Urine Specific Gravity	1.000 1<br>1.005 2<br>1.010 3<br>1.015 4<br>1.020 5<br>1.025 6<br>1.030 7	UR4
Urinalysis Blood	Negative 1<br>Non-haemolysed trace 2<br>Non-haemolysed moderate 3<br>Haemolysed trace 4<br>Small (+) 5<br>Moderate (++) 6<br>Large (+++) 7	UR5
Urine pH	5.0 1<br>6.0 2<br>6.5 3<br>7.0 4<br>7.5 5<br>8.0 6<br>8.5 7	UR6
Urinalysis Protein	Negative 1<br>Trace 2<br>30mg/dL (+) 3<br>100mg/dL (++) 4<br>300mg/dL (+++) 5<br>>2000mg/dL 6	UR7
Urinalysis Nitrite	Negative 1<br>Positive 2	UR8
Urinalysis Leucocytes	Negative 1<br>Trace 2<br>Small (+) 3<br>Moderate (++) 4<br>Large (+++) 5	UR9

For peer review only

BMJ Open

Rationale and Population-based prospective cohort protocol for the Disadvantaged Populations at Risk of Decline in eGFR (CO-DEGREE)

Journal:	*BMJ Open*
Manuscript ID	bmjopen-2019-031169.R1
Article Type:	Protocol
Date Submitted by the Author:	03-Jul-2019
Complete List of Authors:	Gonzalez-Quiroz, Marvin ; National Autonomous University of Nicaragua, Research Centre on Health, Work and Environment<br>Nitsch, Dorothea; LSHTM<br>Hamilton, Sophie ; Imperial College London, School of Public Health, Faculty of Medicine<br>O'Callaghan Gordo, Cristina; Instituto de Salud Global Barcelona, Campus Mar<br>Saran, Rajiv; University of Michigan, Department of Internal Medicine/Nephrology & Epidemiology; University of Michigan<br>Glaser, Jason; La Isla Network<br>Correa-Rotter, Ricardo; National Medical Science and Nutrition Institute Salvador Zubirán, Dept. Nephrology and Mineral Metabolism<br>Jakobsson, Kristina; Goteborgs Universitet,<br>Singh, Ajay; Brigham and Women's Hospital and Harvard medical School<br>Gunawardena, Nalika; World Health Organization Country Office<br>Levin, Adeera; University of British Columbia, Medicine<br>Remuzzi, Giuseppe; IRCCS - Istituto di Ricerche Farmacologiche Mario Negri, Centro Anna Maria Astori, Science and Technology Park Kilometro Rosso<br>Caplin, Ben; University College London Medical School, Centre for Nephrology,<br>Pearce, Neil; London School of Hygiene and Tropical Medicine, Medical Statistics
**Primary Subject Heading**:	Epidemiology
Secondary Subject Heading:	Renal medicine
Keywords:	Prospective cohort study, Chronic kidney disease of unknown aetiology, Generic cohort protocol, Decline in kidney function

SCHOLARONE™
Manuscripts

Rationale and Population-based prospective cohort protocol for the Disadvantaged Populations at Risk of Decline in eGFR (CO-DEGREE)

Marvin Gonzalez-Quiroz, Dorothea Nitsch, Sophie Hamilton, Cristina O´Callaghan-Gordo, Rajiv Saran, Jason Glaser, Ricardo Correa-Rotter, Kristina Jakobsson, Ajay Singh, Nalika Gunawardena, Adeera Levin, Giuseppe Remuzzi, Ben Caplin, Neil Pearce, on behalf of the DEGREE Study Steering Committee

Marvin González-Quiroz, PhD. Research Centre on Health, Work and Environment (CISTA), National Autonomous University of Nicaragua at León (UNAN-León), León, Nicaragua. Centre for Nephrology, University College London, London, UK. Department of Non-Communicable Disease Epidemiology, London School of Hygiene and Tropical Medicine, London, UK. m.quiroz@ucl.ac.uk or marvin99_00@yahoo.es ORCID ID: 0000-0002-0093-6357

Dorothea Nitsch, *Dr.med.* Department of Non-Communicable Disease Epidemiology, London School of Hygiene and Tropical Medicine, London, UK. Dorothea.Nitsch@lshtm.ac.uk

Sophie Hamilton, MSc. School of Public Health, Faculty of Medicine at Imperial College London, London, UK. s.hamilton16@ic.ac.uk

Cristina O´Callaghan-Gordo, PhD. ISGlobal, Barcelona, Spain; Universitat Pompeu Fabra (UPF), Barcelona, Spain; CIBER Epidemiología y Salud Pública (CIBERESP), Madrid, Spain; cristina.ocallaghan@isglobal.org

Rajiv Saran, MD. Division of Nephrology, Department of Internal Medicine and Department of Epidemiology, University of Michigan, Ann Arbor, MIchigan, USA. rsaran@med.umich.edu

Jason Glaser, BSc. La Isla Network, Washington DC, USA. jason@laislanetwork.org

Ricardo Correa-Rotter*,* MD. Department of Nephrology and Mineral Metabolism, National Medical Science and Nutrition Institute Salvador Zubirán, Mexico, DF. correarotter@gmail.com

Kristina Jakobsson*,* MD, PhD. Department of Public Health and Community Medicine, Institute of Medicine, Sahlgrenska Academy, University of Gothenburg, Gothenburg, Sweden.

For peer review only - http://bmjopen.bmj.com/site/about/guidelines.xhtml

Occupational and Environmental Medicine, Sahlgrenska University Hospital, Region Västra Götaland, Gothenburg, Sweden. kristina.jakobsson@amm.gu.se

Ajay Singh, MD. Brigham and Women's Hospital and Harvard medical School, Boston, Massachusetts, USA. Ajay_Singh@hms.harvard.edu

Nalika Gunawardena, MD. World Health Organization Country Office, Colombo, Sri Lanka. gunawardenan@who.int

Levin Adeera, MD. Division of Nephrology UBC, University of British Columbia, ALevin@providencehealth.bc.ca

Giuseppe Remuzzi, *MD.* Instituto di Ricerche Farmacologiche Mario Negri IRCCS, Milan, Italy. giuseppe.remuzzi@marionegri.it

Ben Caplin*, *PhD.* Centre for Nephrology, University College London Medical School, London, UK. b.caplin@ucl.ac.uk

Neil Pearce*, *PhD.* Department of Medical Statistics and Non-Communicable Disease Epidemiology, London School of Hygiene and Tropical Medicine, London, UK, Centre for Global NCDs, London School of Hygiene and Tropical Medicine, London, UK. Neil.Pearce@lshtm.ac.uk

**Equal contribution*

Corresponding author: Marvin Gonzalez-Quiroz

Research Centre on Health, Work and Environment (CISTA), National Autonomous University of Nicaragua at León (UNAN-León), León, Nicaragua

Address: Campus Médico, Facultad de Ciencias Médica, edificio C, León, Nicaragua

Tel: +505 89368376

Email: m.quiroz@ucl.ac.uk or *marvin99_00@yahoo.es*

This **original article** has been seen and approved by all authors listed above and is not under consideration for publication elsewhere.

For peer review only - http://bmjopen.bmj.com/site/about/guidelines.xhtml

Word count for Abstract: 297

Word count for text: 4656

Total word count including tables and figures: 7237

For peer review only

For peer review only - http://bmjopen.bmj.com/site/about/guidelines.xhtml

Abstract

Introduction

A recently recognised form of chronic kidney disease (CKD) of unknown origin (CKDu) is afflicting communities, mostly in rural areas in several regions of the world. Prevalence studies are being conducted in a number of countries, using a standardised protocol, to estimate the distribution of estimated glomerular filtration rate (eGFR), and thus identify communities with a high prevalence of reduced GFR. In this paper, we propose a standardized minimum protocol for cohort studies in high-risk communities aimed at investigating the incidence of, and risk-factors for, early kidney dysfunction.

Methods and analysis

This generic cohort protocol provides the information to establish a prospective population-based cohort study in low-income settings with a high prevalence of CKDu. This involves a baseline survey that included key elements from the DEGREE survey (e.g., using the previously published DEGREE methodology) of a population-representative sample, and subsequent follow-up visits in young adults (without a pre-existing diagnosis of CKD (eGFR<60 mL/min/1.73m^2), proteinuria, or risk factors for CKD at baseline) over several years. Each visit involves a core questionnaire, collection and storage of biological samples. Local capacity to measure serum creatinine (sCr) will be required so that immediate feedback on kidney function can be provided to participants. After completion of follow-up, repeat measures of creatinine should be conducted in a central laboratory, using reference standards traceable to isotope dilution mass

For peer review only - http://bmjopen.bmj.com/site/about/guidelines.xhtml

spectrometry (IDMS) quality control material to quantify the main outcome of eGFR decline over-time, alongside a description of the early evolution of disease and risk factors for eGFR decline.

Ethics and dissemination

Ethical approval will be obtained by local researchers, and participants will provide informed consent before the study commences. Participants will typically receive feedback and advice on their laboratory results, and referral to a local health system where appropriate.

Trial registration number: Not applicable

For peer review only

For peer review only - http://bmjopen.bmj.com/site/about/guidelines.xhtml

Strengths and limitations of this study

- *We propose a prospective generic cohort protocol for populations affected by CKDu in which the sampling frame consists of the entire at-risk population. In addition, the use of this standardised protocol will allow for regional and international comparisons.*
- *Serial eGFR measurements in an apparently healthy population will allow the description of the evolution of disease and reduce problems associated with recall bias and reverse causation when assessing potential risk factors.*
- *Samples will be analysed in a single batch at the end of the study to minimize time-dependent measurement errors.*
- *The use of a standardised protocol will allow for regional and international comparisons.*
- *As for any cohort, loss to follow-up could pose a threat to validity of the study and every effort must be made to mitigate this.*

For peer review only

Introduction

A mysterious form of chronic kidney disease (CKD) is afflicting young adults, mostly in rural communities in a number of low- and middle-income countries.[1-10] This disease has been termed CKD of undetermined cause (CKDu). Several definitions for CKDu exist; the criteria typically include demonstration of renal damage using biomarkers in the absence of diabetes, severe hypertension or evidence of alternative renal diagnoses.[11-14]. This syndrome has caused thousands of deaths and reduced the life expectancy among young adults in Mesoamerica, South Asia, and possibly in other tropical/subtropical regions of the world.[7, 15-19] The cause(s) of CKDu are not yet established, but proposed aetiologies include recurrent dehydration/heat stress, pesticides, infections, and heavy metals.[1, 20-22] In addition, there is no evidence that these forms of CKDu have a unified causality or are due to different aetiologies in diverse parts of the world.

Although a broad range of cross-sectional studies investigating prevalence of CKDu have been conducted in Mesoamerica, South Asia, and other regions of the world,[1-7, 9, 17], these have generally not used standardised methodology, and therefore do not allow for valid international comparisons. A recently published standardised protocol (the Disadvantaged Populations eGFR Epidemiology Study (DEGREE) protocol) for estimating the population distribution of glomerular filtration rate (eGFR), has addressed this concern, and is being used in communities suspected to have a high prevalence of reduced eGFR. The DEGREE protocol makes it possible to undertake comparisons internationally, by mandating a population-representative sample and standardised

For peer review only - http://bmjopen.bmj.com/site/about/guidelines.xhtml

collection of information on sociodemographic factors, occupational and environmental exposures, body composition and kidney function.[23] To date, studies using the DEGREE methodology have been conducted in four countries (Peru, Sri Lanka, India, Malawi), with a number of future projects in preparation or in progress.[17]

A recent meta-analysis highlighted the lack of robust studies that have considered risk factors for early kidney damage in CKDu.[24] This is of key importance as those with even apparently mildly-damaged kidneys (e.g. a borderline elevated serum creatinine but no renal reserve) may experience progressive renal decline in response to a wide-range of exacerbating insults (e.g. episodes of dehydration/heat stress, nephrotoxic medication or other nephrotoxic exposures) making identification of causal associations challenging in those with existing kidney damage. Based on our experience[25, 26] we propose a generic cohort protocol to characterise the decline in kidney function over time and conduct aetiological research in those without pre-existing CKD/risk-factors at baseline but at risk of CKDu. Our focus is on conducting such cohort studies in populations which are at high risk for CKDu i.e., that have previously been classified as such by surveys based on cross-sectional eGFR measurements. In general, this work would follow on from a study using the DEGREE protocol, and hence we will use the term ‘CO-DEGREE’ (cohorts based on the DEGREE study) for such studies. Indeed, in some situations, a DEGREE survey may form the ‘baseline’, with a subgroup of DEGREE survey participants then being selected for follow-up based on age, a single measurement of eGFR ≥60 mL/min/1.73 m^2 (accepting that this is likely a conservative cut-off for pre-existing kidney dysfunction), and without clinical diagnosis or history of hypertension, diabetes mellitus,

For peer review only - http://bmjopen.bmj.com/site/about/guidelines.xhtml

obesity, or other known risk factor that could potentially explain CKD. However, the standardised protocol we propose here can also be used as a 'stand-alone' study design in any well-defined study group, without requiring that a DEGREE survey is conducted first.

We are already conducting such a cohort study in Nicaragua,[25, 26] and have had many challenges to address, including: (i) community engagement, awareness of conditions, political unrest and ethics; (ii) follow-up over time (frequency and minimising loss to follow-up); (iii) fieldwork and laboratory standards to ensure decline is detected; and (iv) regular feedback information on study progress. We will draw on our experience in Nicaragua in presenting both the generic CO-DEGREE protocol, as well as observations on the practical issues involved in conducting such studies in a particular population.

Objectives

Studies using this generic cohort protocol, and contributing to the wider DEGREE collaboration, will aim to:

1. Investigate the evolution of, and risk factors for, kidney function decline over time among populations at risk of CKDu.
2. Compare the evolution, and risk factors for kidney function decline, in different populations and regions at risk of CKDu.
3. Establish a framework for international collaboration and promote a network for future work on the causality of CKDu.

For peer review only - http://bmjopen.bmj.com/site/about/guidelines.xhtml

Rationale for a cohort study of decline in eGFR

A representative sample of those at-risk

Population-based cohort studies have several advantages:[27] Firstly this type of study allows the recruitment of a representative sample of the at-risk population, e.g., it will include workers from a variety of occupations (including unemployed) at the community level. Assuming that the study sample is randomly selected from the entire at-risk population based on a community census, and there are no substantial problems with non-response, these studies are unlikely to be affected by significant selection bias. Furthermore, in contrast to studies conducted solely in an occupational setting, differential loss to follow up is likely to be less problematic, particularly if workers are screened for kidney disease within that setting and potentially denied further work.

Like all prospective cohort studies, to ensure the entire population is 'at-risk', those with the outcome at baseline should be excluded, although it is recognised that investigators may wish to follow-up those with eGFR <60 mL/min/1.73 m^2 and those with established risk factors for CKD for other purposes (see below).

One general disadvantage of population-based studies is that this approach typically requires large sample sizes and long-term follow-up if disease is not highly prevalent. However, the focus of CO-DEGREE is on conducting studies in population with a high prevalence of CKDu (see below).[25, 27]

Handling reverse causation and recall bias

For peer review only - http://bmjopen.bmj.com/site/about/guidelines.xhtml

The problem of reverse-causation (e.g., modification of behaviour or work tasks in response to the diagnosis of renal impairment) can be minimised in a cohort study by focusing on people without pre-existing disease, and then following these initially apparently ‘healthy’ participants over time. Similarly, a cohort approach unlike cross-sectional studies is less prone to recall bias regarding previous exposures.

Measuring kidney function

Quantification of kidney function is most easily undertaken by determining serum creatinine (sCr) concentration, which is relatively easy and cheap to measure, and then calculating the eGFR. A case of CKDu is typically defined by an eGFR <60 mL/min/1.73 m^2 (sustained for at least 3 months to confirm chronicity) in the absence of known causes of kidney disease. However, this dichotomous definition has weaknesses in studies exploring the causation of CKDu, as it is well established that substantial damage may have already occurred at the histological level before serum biomarkers of renal dysfunction become abnormal (and other markers such as proteinuria are often absent in this disease). Furthermore, repeat measures after 3-months are not always performed in cross-sectional surveys, and sCr levels are modified by multiple non-renal factors such as: high animal protein-intake, strenuous exercise, changes in plasma volume, body mass index (BMI), sex, age, ethnicity, and some drugs;[(28)] thus, cross-sectional studies examining associations with reduced eGFR based on a single sCr measurement may be prone to a significant degree of misclassification, especially in smaller studies. Notably, the accuracy of sCr determinations is also an inherent problem (see further below). In

For peer review only - http://bmjopen.bmj.com/site/about/guidelines.xhtml

addition, the CKD-EPI or MDRD equations used to calculate eGFR from sCr,[28] have not been validated in many populations reported to be suffering CKDu,[29] potentially further increasing misclassification bias in cross-sectional studies.

Alternative approaches based on serial eGFR measurements in the same person over time render between-person variation less problematic. If estimated across a period of time using multiple measures with sustained preanalytical and analytical quality, this will also reduce the influence of the within-person factors that are not directly related to kidney damage. In summary, an approach utilising serial eGFR measures substantially improves the potential to identify risk/causal factors for CKDu as well as allowing the description of the evolution of disease.

Core protocol

Study design

This is a prospective cohort study protocol for studying decline in kidney function over time in populations with high reported prevalence of CKDu, primarily in low- and middle-income countries (LMICs). We consider the following study design issues: (i) population sampling strategy, and follow-up interval (ii) questionnaire development and delivery, (iii) clinical measurements and biosampling, and (iv) data management and reporting.[25] (See Figure 1) In addition, we discuss: (a) sample size and follow-up duration; and (b) ethical considerations.

For peer review only - http://bmjopen.bmj.com/site/about/guidelines.xhtml

Population, sampling strategy and follow-up interval

In Mesoamerica, CKDu typically affects young men on the Pacific Coast. This population is dying in their 40s, often younger, from end stage renal disease.[15, 30] The disease appears to occur at a later age in South Asia, with few cases occurring in men in their 20s.[7, 31] Nevertheless, one might expect preliminary changes in GFR to occur early in adulthood. In general, the study population should include participants who are old enough to experience an identifiable decline in kidney function, but not older age-groups (e.g., >60 year-old) where the prevalence of CKD is already high in many populations globally (e.g. up to 10%). Thus, inclusion criteria should be tailored to the local disease profile, but the default approach should be to recruit participants aged 18-40 years-old (though 18-30 might be more appropriate in Central America, and 18-50 may be more appropriate in areas such as South Asia where age of onset appears older). The rationale for including people ≥18-year-old was based on definition on adult life, and may be lowered, especially in populations where the working life starts years earlier. A population-census should be conducted to identify all potential participants in the appropriate age range and either the entire population recruited, or a random sample selected. In either case, response rates by age and sex, should be reported.

The focus of these studies is to conduct aetiological research in those without traditional CKD/risk-factors at baseline, thus, the sample size estimates (see below) are based on following a cohort in which those with evidence of pre-existing CKD, diabetes or hypertension have been excluded.[25] Diabetes can be diagnosed by self-report, use of

For peer review only - http://bmjopen.bmj.com/site/about/guidelines.xhtml

medication, or lab tests (fasting serum glucose: ≥7.0 mmol/l or HbA1C ≥48mmol/mol),[32, 33] and hypertension by self-report, use of medication or measurement (seated, average BP ≥140/90mmHg on second and third of three readings).[34]

In addition to self-report of CKD, those with previously detected eGFR<60 mL/min/1.73m^2, proteinuria, (e.g. albumin/creatinine ratio, ACR, >300mg/g or dipstick 3+ or greater)[35] on testing at baseline should be excluded from the study. It is recognised that a proportion of participants not excluded by these criteria may still have some form of underlying kidney abnormality (e.g. low-level proteinuria), and some of those excluded due to a low eGFR at baseline may go on to recover function, but this represents a pragmatic approach to excluding those with significant pre-existing renal disease at baseline. Furthermore, for practical, ethical or scientific reasons (for example, to gain insight into progression of established CKDu or other non-communicable disease research aims), investigators may wish to study an entire population (including those with pre-existing clinical diagnosis of, or newly identified, CKD, diabetes mellitus, and hypertension), but in that case, it is important to ensure that there are sufficient 'disease free' participants included at baseline to meet the sample size requirements (see Table 1). Although the disease is generally more common in men, women with CKDu are of strong scientific interest in that they may suggest alternative risk factors, or help to rule out some that have been previously proposed. Hence recruitment should in general involve equal numbers of males and females, though women who are pregnant at recruitment are also excluded, since pregnancy-related changes in eGFR are challenging to interpret.

For peer review only - http://bmjopen.bmj.com/site/about/guidelines.xhtml

The baseline study visit will require the administration of the core-questionnaire, with additional context-specific additions, clinical measurements and biological samples. Subsequent to the baseline visit, follow-up visits should be conducted at least annually for a minimum follow-up of two-years to evaluate the study outcome and keep close contact with the participants and update their contact information. This will help minimize the loss to follow-up at each study point. Substantial seasonal variation in eGFR has been reported in a number of settings (both CKDu related and unrelated).[26, 36-38] Therefore, the conduct of additional study visits at a 6-monthly interval (e.g. at beginning and end of summer season) might be useful in explaining within-person eGFR variation as well as providing important information for the wider population on the significance of kidney function testing at different time point in the year (perhaps for a subset of participants or a proportion of the follow-up period). (See Table 2)

Questionnaires

The purpose of the baseline core-questionnaire is to obtain a minimum dataset to explore associations with decline in kidney function and make comparisons within and between persons. The baseline core-questionnaire (supplementary file 1) is based on the questionnaire used in the DEGREE protocol and has been used in DEGREE-related studies in a number of settings. The baseline core-questionnaire represents a minimum data set and it will provide basic information on exposures such as sociodemographic factors, occupational and environmental exposure, lifestyle, diagnosis of infectious

For peer review only - http://bmjopen.bmj.com/site/about/guidelines.xhtml

diseases, and medication. Local research teams may decide to add data items of specific interest to the core dataset, particularly items of relevance to societal and occupational context and/or environmental samples. They also have the responsibility to translate, validate, and to make any local contextual changes. Training procedures for the field-staff should be documented.

Researchers will return to field (at least) annually for in-person follow-up visits. At these follow-up visits participants are invited to respond a follow-up questionnaire (supplementary file 2), provide biosamples and update their contact information.

Clinical measurements

Blood pressure and heart rate should be measured on the right arm after 5 minutes rest in the sitting position using an automated sphygmomanometer, WHO validated for the clinical setting (example: Omron HEM-907XL sphygmomanometer) and the average of the second and third of three readings recorded. Subjects height and weight (in centimetres and kilograms) should be measured (without shoes) using a stadiometer and digital calibrated scales.

Biosamples

Fasting blood and urine samples will be collected at each study visit and stored in the field into coolers with icebox (4ºC) no more than 4 hours before processing.

For peer review only - http://bmjopen.bmj.com/site/about/guidelines.xhtml

Dipstick urinalysis should be performed by using electronic readers (urine chemistry analyser) where possible, or otherwise at least 10% of tests should be re-analysed by a second investigator. Parameters that should be reported are: urinary specific gravity, pH, protein, blood, leucocytes, nitrite, glucose, etc. Investigators with access to ACR measurements may wish to perform these assays (at least at baseline and annually).

Samples for serum analysis should be centrifuged at 3500 rpm for 10 minutes within 4 hours of collection, and subsequently separated into at least four aliquots of 1-2 mL and stored at ≤-20ºC (ideally -80ºC). One aliquot should be used for contemporary sCr measurements e.g. by using the modified Jaffe assay (ideally also using standards traceable to isotope dilution mass spectrometry [IDMS] reference material). At baseline and during each study visit a cross-checking of local lab quality control is highly recommended to ensure that sCr determinations are comparable as these lab results may guide referral to clinical care for participants during the follow-up period. A further aliquot should be stored for a repeat batch measurement of sCr in all samples (a subset of samples from each study visit will be adequate if IDMS referenced methods are used on initial measurement) at the end of follow-up using a method traceable to an IDMS reference material (and potentially also cystatin C).

The CO-DEGREE group suggest the storage of at least a further two 1-2mL aliquots of serum and a similar amount of urine in addition to those described above. Additional samples and analyses should be pursued depending on the priorities of the local research team. All samples for future analysis should be stored at ≤-20ºC (ideally -80ºC) in a local

For peer review only

or international biobank. Such a biobank requires an uninterruptible power supply to protect the samples.

Investigators should assess (as part of their public engagement efforts), and if appropriate, obtain consent from participants for future use of samples for further (specific and/or more general) use both locally and internationally (e.g. through the DEGREE collaboration) as well as ensure that storage capacity is available.

Data management and reporting

Questionnaires and samples will be labelled using a unique bar-code to maintain participant confidentiality. Electronic data capture systems such as Open Data Kit [(39)] may be the most resource efficient method to capture questionnaire data but where hard-copies are used double data-entry should be undertaken to minimise the transcription errors.

The CO-DEGREE protocols are openly available to interested research teams. Although primarily designed to be used in population-based studies similar approaches could also be used in an occupational or other selected cohorts.

Each centre will be ‘owner’ of their data and expected to publish the results of their study independently. However, where a study is registered as part of the DEGREE collaboration the coordinating centre will request a digital copy of anonymized individual-level data to allow the undertaking of international comparisons. In addition, a summary of local

For peer review only - http://bmjopen.bmj.com/site/about/guidelines.xhtml

contextual information and a description of the population characteristics along with response rates will be requested. The importance of such information is emphasized.

Sample size and follow-up duration

The overall size of the cohort will be largely dependent on the proportion of the 'healthy' population which is expected to experience a 'substantial' decline in eGFR over time in the community as a result of CKDu. As discussed above, demonstrating that reduced renal function without diabetes, hypertension, or known kidney diseases is prevalent on a cross-sectional basis is a necessary first step before pursuing this work. If for example this study protocol was to be conducted in a general population sample in Europe or the USA with similar exclusion criteria, there would be very little or no decline of kidney function in the young adult population. In contrast, in our Nicaragua study of apparently healthy adults aged 18-30 years,[25, 26] there was a clearly distinct subgroup which experienced a marked decline in kidney function over a short time, whereas the eGFR in the other study participants was relatively stable. Given this distribution of such eGFR trajectories in the population we would expect any analysis of risk factors to be conducted using a prospective case-control approach.

Therefore, the sample size requirements to detect an association with an exposure at any given power will be determined by the following factors:

1. Proportion of the population that experience 'substantial' decline

   In turn the power to detect 'substantial' decline will depend on:

   a) The rate of eGFR decline in those affected

For peer review only - http://bmjopen.bmj.com/site/about/guidelines.xhtml

b) The duration of follow-up

c) The number of eGFR measures

2. Proportion of general population exposed to any exposure of interest
3. Effect size of any exposure
4. The study retention rate

Taking a simplistic approach, the duration of the study should be designed so that those affected have sustained a clinically important loss of kidney function, e.g. 20% of normal eGFR. Therefore, if CKDu in the study population is predicted, from a baseline of ≥60ml/min eGFR, to lead to a loss of eGFR of a magnitude of 5% each year (~7mL/min/1.73 m^2/year) the study duration should be 4 years. If alternatively, loss is predicted to be 10% each year study duration could be as short as 2 years. Additional eGFR measures, over and above the suggested annual frequency will reduce error associated with determining trajectory (and might be performed for the reasons discussed above) but either way a minimum follow-up of 2 years is recommended.

After basing the study duration on the expected rate of eGFR decline among those affected, the sample size can then be calculated on the basis of the expected frequency of ‘substantial’ decline amongst the population and the effect size of any proposed exposure that it is desirable to detect. A number of scenarios are outlined in Table 1. A further (e.g. 20%, depending on local circumstances) increase in target recruitment is advised to allow for loss to follow-up.

For peer review only - http://bmjopen.bmj.com/site/about/guidelines.xhtml

Finally, these initial sample sizes will need adjustment for exclusions based on estimates of the prevalence of previously unknown CKD (based on eGFR/albuminuria tests), diabetes, hypertension or other known causes of CKD at baseline (unless these data are already available from a previously conducted cross-sectional study). It is worth considering whether people who may have CKD (or CKD risk factors) will be aware of this, as this may affect the numbers of participants that will be retained for the analysis following testing. For example, if there is screening for kidney problems (as in some Central American Sugarcane mills or community-based screening in Sri Lanka), then potential cohort participants may be aware of their kidney function status and can be excluded from the study sample prior to recruitment. For example, 5% of the target population in the community studied in Nicaragua reported pre-existing CKD. Nevertheless, there was an additional 10% who had undiagnosed impaired kidney function at baseline assessment based on their laboratory findings, highlighting the importance of identifying an age-group where CKDu is not already highly prevalent so as to satisfy a key inclusion criterion (absence of CKD at baseline) when calculating sample sizes.

Ethics and dissemination

Local research teams will ensure these studies are conducted in accordance with the Declaration of Helsinki Principles and be responsible for assuring that the work is approved by the local institutional review board (IRBs). Written informed consent will be obtained from all participants before taking part in the study. Information should be

For peer review only - http://bmjopen.bmj.com/site/about/guidelines.xhtml

transparent in terms of using the data and biosamples stored for future research. Typically, a key aspect of the ethical review of any protocol is a discussion surrounding the provision of feedback and advice to participants when abnormal results become available. In most settings these processes should be developed in partnership with local communities. Furthermore, mechanisms will need to be established in collaboration with local health providers/healthcare systems to define pathways for participants needing referral for medical care. Findings from these studies should be disseminated widely by publication in peer-reviewed journals and presentations/representations to relevant local stake holders.

Patient and public involvement

Patients or member of the public were not involved in the design of this protocol. Procedures will vary by location however the DEGREE Steering Committee would encourage active involvement of lay members of study communities in additional design elements and implementation of these studies particularly relating to the ethical issues above. For example, it is expected that study participants will receive the results of their lab tests, explanations of them and a reference to the relevant health centre if appropriate. However, the best mechanisms for doing this will vary by location.

Experience with the CO-DEGREE protocol in Nicaragua

The protocol presented here is, by necessity, generic. The approaches and challenges of implementing the protocol will vary widely in different populations and regions of the world. However, since we have already implemented this protocol in a study in

For peer review only - http://bmjopen.bmj.com/site/about/guidelines.xhtml

Nicaragua,[25, 26] we will make some observations on the practicalities, and challenges, or implementing the protocol in this context.

The Nicaragua study involved community-based follow-up in Leon and Chinandega departments.[25] A number of strategies were used to maximise response and retention rates. As the workday starts very early in the morning and finishes late in the afternoon attempts were made to conduct data collection during economically less active (e.g. each side of the main sugar harvest) periods of the year, so as to still capture approximately 30% of participants who were employed at the time. Additionally, participants receive their kidney test results within a fortnight of the study visits and receive reimbursement of expenses and any lost income they have incurred to attend the study visit. Although study visits have been timetabled to occur outside of the harvest season, employees still express the concern that their employment opportunities might be affected by taking part in the study. In an attempt to mitigate against these types of consequences, the study team have corresponded with local employers explaining the content and extent of this study in order to reduce any concerns about workers' participation. In addition, the study team takes particular precautions to maintain participant's confidentiality during the study and beyond.

Conducting a follow up study in a rural area remains a major challenge. Alongside the logistical challenges of reaching geographically isolated neighbourhoods along poor quality roads, a significant obstacle has been internal and external migration due to lack

For peer review only - http://bmjopen.bmj.com/site/about/guidelines.xhtml

of employment source or social unrest. Rural communities have a tradition of working with seasonal crops and sugarcane workers often leave their communities at the end of each harvest season, to go abroad or to other regions within the country in search of temporary employment. In our study, at the end of each harvest, up to 30% of the study population had left their communities in search of alternative employment during the non-harvest period in our study. Despite these problems our team achieved attendance at 92% of all scheduled visits over two years.[25, 26] However the level of investment of time and resources should not be underestimated.

Finally, continuing community engagement and the maintenance of good relationships between researchers, community leaders, participants and communication with local health care system have been key. The development of standardised procedures for use by the research team may be useful in this context, e.g., a reference flowchart for communication with local health posts/primary hospital or hospital for persons with health problems detected during the study.

Discussion

The CO-DEGREE protocol was developed in response to the highly prevalent form of CKD of unknown cause that is affecting Mesoamerica and other countries around the globe. To date, the existing epidemiological studies of CKDu have provided an incomplete understanding of the evolution of and risk factors for disease. This CO-DEGREE protocol aims to provide a framework to address this.

For peer review only - http://bmjopen.bmj.com/site/about/guidelines.xhtml

This CO-DEGREE protocol is designed to capture the entire at-risk population by aiming to recruit men and women, and those that work across a variety of different occupations. The main outcome measure of within-person loss of eGFR over time, which means it is should be possible to capture the earliest disease stages of disease, making associations with possible causal exposures (and exacerbating factors) less prone to reverse causation and recall bias.

We do not underestimate the challenges posed by the lack of language-validated and standardized exposure questionnaires in this area. The accompanying questionnaire represents a minimum and most studies will utilise an expanded dataset. Currently there is an absence of globally generalizable instruments to capture environmental and occupational exposures, however the DEGREE group is undertaking further work in this area. Additionally, short or long-term environmental measurements and/or novel biomarkers that capture exposure to heat, agrichemicals, and/or infection in either the community or workplace are likely to be valuable additions to this type of study but are beyond the scope of this basic protocol.

Finally, it should be emphasized that this protocol is not suitable for studying the progression of CKD in general, due to the specific constraints introduced by excluding those with hypertension, diabetes and CKD as well as other known causes of CKD (i.e. those with proteinuria and/or with reduced eGFR) at baseline. Indeed, in settings where there is not a high prevalence of CKDu, a cohort comprised of people without traditional

For peer review only - http://bmjopen.bmj.com/site/about/guidelines.xhtml

risk factors for CKD or with CKD would be unlikely to identify any detectable kidney function loss over time in the young-adult population. For studies outside the CKDu arena, investigators are advised to use alternative methodologies using established protocols, for example, the CRIC study.[40]

In conclusion, we have designed a CO-DEGREE protocol that can be used in the different settings around the globe to investigate the evolution of CKDu and associated risk factors for decline in kidney function. These studies should provide important information on the early decline in kidney function across different affected areas as well as key insight into the cause(s) of disease.

For peer review only

For peer review only - http://bmjopen.bmj.com/site/about/guidelines.xhtml

Reference

1. Wegman D, Crowe J, Hogstedt C, Jakobsson K, Wesseling C, editors. Mesoamerican nephropathy: report from the second international research workshop on MeN. Heredia, C.R: SALTRA/IRET-UNA; 2016. Report No.: ISBN 978-9968-924-33-7.
2. Torres C, Aragon A, Gonzalez M, Lopez I, Jakobsson K, Elinder CG, et al. Decreased kidney function of unknown cause in Nicaragua: a community-based survey. Am J Kidney Dis. 2010;55(3):485-96.
3. O'Donnell JK, Tobey M, Weiner DE, Stevens LA, Johnson S, Stringham P, et al. Prevalence of and risk factors for chronic kidney disease in rural Nicaragua. Nephrol Dial Transplant. 2011;26(9):2798-805.
4. Orantes CM, Herrera R, Almaguer M, Brizuela EG, Hernandez CE, Bayarre H, et al. Chronic kidney disease and associated risk factors in the Bajo Lempa region of El Salvador: Nefrolempa study, 2009. MEDICC Review. 2011;13(4):14-22.
5. Orantes CM, Herrera R, Almaguer M, Brizuela EG, Nunez L, Alvarado NP, et al. Epidemiology of chronic kidney disease in adults of Salvadoran agricultural communities. MEDICC Review. 2014;16(2):23-30.
6. Jayasekara JM, Dissanayake DM, Adhikari SB, Bandara P. Geographical distribution of chronic kidney disease of unknown origin in North Central Region of Sri Lanka. Ceylon Med J. 2013;58(1):6-10.
7. Jayatilake N, Mendis S, Maheepala P, Mehta FR, Team CKNRP. Chronic kidney disease of uncertain aetiology: prevalence and causative factors in a developing country. BMC Nephrol. 2013;14:180.
8. Ganguli A. Uddanam Nephropathy/Regional Nephropathy in India: Preliminary Findings and a Plea for Further Research. Am J Kidney Dis. 2016;68(3):344-8.
9. Jayasumana C, Orantes C, Herrera R, Almaguer M, Lopez L, Silva LC, et al. Chronic interstitial nephritis in agricultural communities: a worldwide epidemic with social, occupational and environmental determinants. Nephrol Dial Transplant. 2017;32(2):234-41.
10. Peraza S, Wesseling C, Aragon A, Leiva R, Garcia-Trabanino RA, Torres C, et al. Decreased kidney function among agricultural workers in El Salvador. Am J Kidney Dis. 2012;59(4):531-40.
11. García-Trabanino R, Cerdas M, Madero M, Jakobsson K, Barnoya J, Crowe J, et al. Nefropatía mesoamericana: revisión breve basada en el segundo taller del Consorcio para el estudio de la Epidemia de Nefropatía en Centroamérica y México (CENCAM). Nefrología Latinoamericana. 2017;14(1):39-45.
12. Lozier M, Turcios-Ruiz RM, Noonan G, Ordunez P. Chronic kidney disease of nontraditional etiology in Central America: a provisional epidemiologic case definition for surveillance and epidemiologic studies. Rev Panam Salud Publica. 2016;40(5):294-300.
13. Rajapakse S, Shivanthan MC, Selvarajah M. Chronic kidney disease of unknown etiology in Sri Lanka. Int J Occup Environ Health. 2016;22(3):259-64.
14. WHO. Workshop report: Designing a step-wise approach to estimate the burden and to understand the etiology of CKDu in Sri Lanka. Sri Lanka: WHO; 2016, 24-25th October.

For peer review only - http://bmjopen.bmj.com/site/about/guidelines.xhtml

15. Ordunez P, Nieto FJ, Martinez R, Soliz P, Giraldo GP, Mott SA, et al. Chronic kidney disease mortality trends in selected Central America countries, 1997-2013: clues to an epidemic of chronic interstitial nephritis of agricultural communities. J Epidemiol Community Health. 2018;72(4):280-6.
16. Ordunez P, Saenz C, Martinez R, Chapman E, Reveiz L, Becerra F. The epidemic of chronic kidney disease in Central America. Lancet Glob Health. 2014;2(8):e440-1.
17. Ekiti ME, Zambo JB, Assah FK, Agbor VN, Kekay K, Ashuntantang G. Chronic kidney disease in sugarcane workers in Cameroon: a cross-sectional study. BMC Nephrol. 2018;19(1):10.
18. Ministry of Health Nutrition and Indigenous Medicine - Medical Statistics Unit. Annual health bulletin of Sri Lanka 2015. Sri Lanka: Ministry of Health, Nutrition and Indigenous Medicine; 2017 [cited 2017 June 20]. Available from: http://www.health.gov.lk/moh_final/english/public/elfinder/files/publications/AHB/2017/AHB%202015.pdf.
19. Nanayakkara S KT, Rajapurkar MM, John GT, Kirpalani AL. . What do we know about chronic kidney disease in India: first report of the Indian CKD registry. . BMC Nephrol 2012;13(10).
20. Correa-Rotter R, Wesseling C, Johnson RJ. CKD of unknown origin in Central America: the case for a Mesoamerican nephropathy. Am J Kidney Dis. 2014;63(3):506-20.
21. Wesseling C, Crowe J, Hogstedt C, Jakobsson K, Lucas R, Wegman DH. The epidemic of chronic kidney disease of unknown etiology in Mesoamerica: a call for interdisciplinary research and action. Am J Public Health. 2013;103(11):1927-30.
22. Wesseling C, Crowe J, Hogstedt C, Jakobsson K, Lucas R, Wegman D, et al. First international research workshop on mesoamerican nephropathy (MeN). Heredia, C.R.: SALTRA/IRET-UNA; 2013. Report No.: ISBN 978-9968-924-06-1.
23. Caplin B, Jakobsson K, Glaser J, Nitsch D, Jha V, Singh A, et al. International Collaboration for the Epidemiology of eGFR in Low and Middle Income Populations - Rationale and core protocol for the Disadvantaged Populations eGFR Epidemiology Study (DEGREE). BMC Nephrol. 2017;18(1):1.
24. Gonzalez-Quiroz M, Pearce N, Caplin B, Nitsch D. What do epidemiological studies tell us about chronic kidney disease of undetermined cause in Meso-America? A systematic review and meta-analysis. Clin Kidney J. 2018;11(4):496-506.
25. Gonzalez-Quiroz M, Camacho A, Faber D, Aragon A, Wesseling C, Glaser J, et al. Rationale, description and baseline findings of a community-based prospective cohort study of kidney function amongst the young rural population of Northwest Nicaragua. BMC Nephrol. 2017;18(1):16.
26. Gonzalez-Quiroz M, Smpokou ET, Silverwood RJ, Camacho A, Faber D, Garcia BR, et al. Decline in Kidney Function among Apparently Healthy Young Adults at Risk of Mesoamerican Nephropathy. J Am Soc Nephrol. 2018;29(8):2200-12.
27. Caplin Ben, González-Quiroz Marvin, Pearce Neil. Gaining insights into the evolution of CKDnt from community-based follow up studies. In: SALTRA, editor. Second International Workshop on Mesoamerican Nephropathy; San José. Costa Rica: SALTRA; 2015.

For peer review only - http://bmjopen.bmj.com/site/about/guidelines.xhtml

28. Padala S, Tighiouart H, Inker LA, Contreras G, Beck GJ, Lewis J, et al. Accuracy of a GFR estimating equation over time in people with a wide range of kidney function. Am J Kidney Dis. 2012;60(2):217-24.
29. Inker LA, Schmid CH, Tighiouart H, Eckfeldt JH, Feldman HI, Greene T, et al. Estimating glomerular filtration rate from serum creatinine and cystatin C. N Engl J Med. 2012;367(1):20-9.
30. Garcia-Trabanino R, Trujillo Z, Colorado AV, Magana Mercado S, Henriquez CA, En nombre de la Asociacion de Nefrologia e Hipertension Arterial de El S. Prevalence of patients receiving renal replacement therapy in El Salvador in 2014. Nefrologia. 2016;36(6):631-6.
31. Jha V, Garcia-Garcia G, Iseki K, Li Z, Naicker S, Plattner B, et al. Chronic kidney disease: global dimension and perspectives. Lancet. 2013;382(9888):260-72.
32. American Diabetes A. 2. Classification and Diagnosis of Diabetes: Standards of Medical Care in Diabetes-2018. Diabetes Care. 2018;41(Suppl 1):S13-S27.
33. Association American D. Updates to the Standards of Medical Care in Diabetes-2018. Diabetes Care. 2018;41(9):2045-7.
34. Chobanian AV, Bakris GL, Black HR, Cushman WC, Green LA, Izzo JL, Jr., et al. Seventh report of the Joint National Committee on Prevention, Detection, Evaluation, and Treatment of High Blood Pressure. Hypertension. 2003;42(6):1206-52.
35. KDIGO. KDIGO 2012 Clinical Practice Guideline for the Evaluation and Management of Chronic Kidney Disease. Kidney Int Supps. 2013;3(1):1-150.
36. Wesseling C, Aragon A, Gonzalez M, Weiss I, Glaser J, Bobadilla NA, et al. Kidney function in sugarcane cutters in Nicaragua--A longitudinal study of workers at risk of Mesoamerican nephropathy. Environ Res. 2016;147:125-32.
37. Wesseling C, Aragon A, Gonzalez M, Weiss I, Glaser J, Rivard CJ, et al. Heat stress, hydration and uric acid: a cross-sectional study in workers of three occupations in a hotspot of Mesoamerican nephropathy in Nicaragua. BMJ Open. 2016;6(12):e011034.
38. Laws RL, Brooks DR, Amador JJ, Weiner DE, Kaufman JS, Ramirez-Rubio O, et al. Changes in kidney function among Nicaraguan sugarcane workers. Int J Occup Environ Health. 2015;21(3):241-50.
39. Open Data KIT. Longitudinal Clinic Study App: GitHub, Inc; 2018 [cited 2018 July 28]. Available from: https://opendatakit.org/use/2_0_tools/odk-application-designer-2-0-rev126/.
40. Feldman HI, Appel LJ, Chertow GM, Cifelli D, Cizman B, Daugirdas J, et al. The Chronic Renal Insufficiency Cohort (CRIC) Study: Design and Methods. J Am Soc Nephrol. 2003;14(7 Suppl 2):S148-53.

For peer review only

Acknowledgments

The authors wish to thank all DEGREE Steering Committee members.

DEGREE Study Steering Committee

Neil Pearce (UK) (Chair)
Ben Caplin (UK) (Co-chair)
Jason Glaser (USA)
Ricardo Correa-Rotter (Mexico)
Kristina Jakobsson (Sweden)
Ajay Singh (USA/India)
Antonio Bernabe-Ortiz (Peru)
Emmanuel Burdmann (Brazil)
Marvin Gonzalez (Nicaragua)
Vivekanand Jha (India)
Rick Johnson (USA)
Phabdheep Kaur (India)
Pronpimolk Kongtip (Thailand)
Hans Kromhout (Netherlands)
Adeera Levin (Canada)
Magdalena Madero Rovalo (Mexico)
Dorothea Nitsch (UK)
Moffat Nyirenda (Ugand/Malawi)
Cristina O'Callaghan-Gordo (Spain)
Pablo Perel (UK/Argentina)
Dorairaj Prabhkaran (India)
Narayan Prasad (India)
Giuseppe Remuzzi (Italy)
Rajiv Saran (USA)
Liam Smeeth (UK)
Vidhya Venugopal (India)

Observers
Nalika Gunawardenan (Sri Lanka)

For peer review only

Authors´contributions

The CO-DEGREE protocol was conceived by MGQ, BC, DN, and NP. Design of the study and drafting the protocol was done by MGQ, BC, DN, NP, SH, COG, RS, JG, RCR, KJ, AS, NG, AL, and GR. All authors contributed to and approved the final manuscript.

Funding statement

This work was supported by grants from the UK Colt Foundation and the UK Medical Research Council (MR/P02386X/1).

Competing interests statement

The authors declare they have not competing interests

Patient consent for publication

Not required

Data sharing statement

Please contact the relevant authors for data requests or any additional information

For peer review only

List of figures and tables

Figures:

Figure 1: Flow chart and study procedures of CO-DEGREE protocol

Tables:

Table 1: Sample Size Calculations

Table 2. Details and procedures of the baseline study visit and subsequence follow-up.

Supplementary material

CO-DEGREE baseline questionnaire

CO-DEGREE follow-up questionnaire

For peer review only - http://bmjopen.bmj.com/site/about/guidelines.xhtml

Table 1: Sample Size Calculations

Parameters	Scenario 1	Scenario 2	Scenario 3	Scenario 4	Scenario 5	Scenario 6	Scenario 7	Scenario 8
**Population frequency of eGFR decline**	0.04	0.06	0.08	0.10	0.04	0.06	0.08	0.10
**Proportion population exposed**	0.5							
**Odds ratio associated with exposure**	2				3			
**P (outcome|unexposed)**	0.027	0.04	0.053	0.066	0.02	0.03	0.04	0.05
**P (outcome|exposed)**	0.054	0.08	0.106	0.132	0.06	0.09	0.12	0.15
**Group size**	993	686	405	436	463	317	243	200
**Sample size**	**1986**	**1372**	**810**	**872**	**926**	**634**	**486**	**400**

Abbreviations, eGFR, estimated glomerular filtration rate; P: probability. Assumes 1-**β**=0.80; =0.05; Calculations based on equal proportion of the population exposed/unexposed for simplicity. No adjustments made for loss to follow-up or multiple testing.

For peer review only

For peer review only - http://bmjopen.bmj.com/site/about/guidelines.xhtml

Table 2. Details and procedures of the baseline study visit and subsequence follow-up.

Items	***Baseline visit (0 month)***	***Follow-up period (variable)***				
		12 months	*24 months*	*36 months*	*48 months*	*At completion*
Community census	X	-	-	-	-	-
Participants enrolment	X	-	-	-	-	-
Informed consent	X					
Update personnel contact information	X	X	X	X	X	
Anthropometric measurements	X	X	X	X	X	
Biological samples	X	X	X	X	X	
Baseline core-questionnaire	X	-	-	-	-	
Follow-up questionnaire		X	X	X	X	
Local serum creatinine measurement	X	X	X	X	X	
Results feedback	X	X	X	X	X	
Biobank	X	X	X	X	X	
Batch testing of serum creatinine						X

For peer review only - http://bmjopen.bmj.com/site/about/guidelines.xhtml

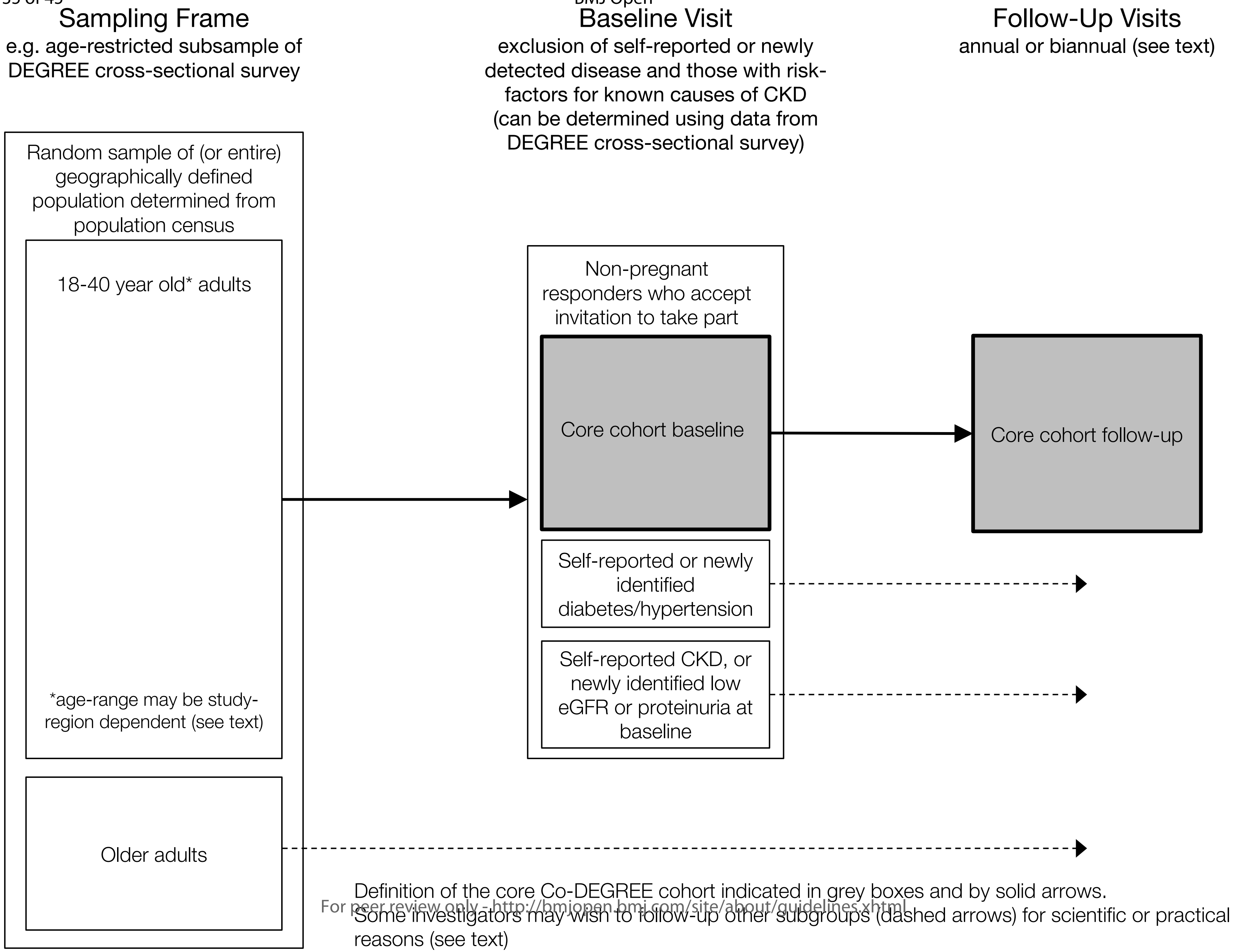

Definition of the core Co-DEGREE cohort indicated in grey boxes and by solid arrows.
Some investigators may wish to follow-up other subgroups (dashed arrows) for scientific or practical reasons (see text)

For peer review only - http://bmjopen.bmj.com/site/about/guidelines.xhtml

CO-DEGREE: baseline questionnaire

PASTE THE ID LEVEL HERE

Participant Identification Number |__|__|__| |__|__|__| |__|__|__|

CO-DEGREE Basic Core Questionnaire

	Response	Code
Study site ID	|__|__|__|	I1
Interviewer ID	|__|__|__|	I2
Study visit number	|__|__|__|	I3
Date of completion of the instrument	|__|__| dd |__|__| mm |__|__|__|__| year	I4

Consent, Interview Language and Name	Response	Code
Consent has been read and obtained	Yes 1<br>No 2 If NO, END	I5
Interview Language *[Insert Language]*	English 1<br>*[Add others]* 2<br>*[Add others]* 3<br>*[Add others]* 4	I6
Time of interview<br>(24 hour clock)	|__|__| : |__|__| hrs mins	I7
Family Surname		I8
First Name		I9
Address:		
Additional Information that may be helpful		
Contact phone number where possible		I10

For peer review only - http://bmjopen.bmj.com/site/about/guidelines.xhtml

CO-DEGREE: baseline questionnaire

PASTE THE ID LEVEL HERE

Participant Identification Number |__|__|__| |__|__|__| |__|__|__|

CO-DEGREE basic core clinical and anthropometry measurements

Question	Response	Code
Ambient Temperature<br>(at time of examination measured in shade)	|__|__|__| °C	Temp
**Blood Pressure**		
Question	Response	Code
Interviewer ID	|__|__|__|	M1
Device ID for blood pressure	|__|__|__|	M2
Cuff size used	Small 1<br>Medium 2<br>Large 3	M3
Reading 1	Systolic (mmHg) |__|__|__|	M4a
	Diastolic (mmHg) |__|__|__|	M4b
	Heart rate |__|__|__|	M4c
Reading 2	Systolic (mmHg) |__|__|__|	M5a
	Diastolic (mmHg) |__|__|__|	M5b
	Heart rate |__|__|__|	M5c
Reading 3	Systolic (mmHg) |__|__|__|	M6a
	Diastolic (mmHg) |__|__|__|	M6b
	Heart rate |__|__|__|	M6c
During the past two weeks, have you been treated for raised blood pressure with drugs (medication) prescribed by a doctor or other health worker?	Yes 1<br>No 2	M7
**Height, and Weight**		
For women: Are you pregnant?	Yes 1 if yes should be excluded<br>No 2	M8
Have you eaten yet today?	Yes 1<br>No 2	M9
Interviewer ID	|__|__|__|	M10
Height	in Centimetres (cm) |__|__|__|	M11
Weight<br>*If too large for scale 666.6*	in Kilograms (kg) |__|__|__|	M12

For peer review only - http://bmjopen.bmj.com/site/about/guidelines.xhtml

CO-DEGREE: baseline questionnaire

PASTE THE ID LEVEL HERE

Participant Identification Number |_|_|_|_| |_|_|_|_| |_|_|_|_|

CO-DEGREE basic core questionnaire (adapted from DEGREE PROTOCOL)

Question	Response	Code
Sex (*Record Male / Female as observed*)	Male 1<br>Female 2	C1
What is your date of birth?<br>*Don't Know 77 77 7777*	dd mm year	C2
How old are you?	Years |_|_|_|	C3
In total, how many years have you spent at school and in full-time study (excluding pre-school)?	Years |_|_|_|	C4
What is your *[insert relevant ethnic group / racial group / cultural subgroup / others]* background?	*[Locally defined]* 1<br>*[Locally defined]* 2<br>*[Locally defined]* 3<br>Refused 88	C6
Which of the following best describes your main work status over the past 12 months?	Government employee 1<br>Non-government employee 2<br>Self-employed 3<br>Non-paid 4<br>Student 5<br>Homemaker 6<br>Retired 7<br>Unemployed (able to work) 8<br>Unemployed (unable to work) 9<br>Unpaid domestic 10<br>Refused 88	C8
If you are working what is your main occupation [FREE TEXT]:		OCCTXT
What task do you perform? [FREE TEXT]		TASKTXT
How many years have you been working in your current job?	Years |_|_|_|	C9
How many hours do you work daily?	Hours |_|_|_|	C10
Where do you work mostly?	Indoors 1<br>Outdoors 2<br>Both 3	C11
Do you take work breaks in shade?	Yes 1<br>No 2	C12
Do you work in a very hot working environment?	Seldom or never 1<br>Few times 2<br>Regularly 3<br>Frequently 4<br>Always or almost always 5	C13
How much physical effort did you do at work?	Slight effort 1<br>Moderate effort 2<br>Hard effort 3<br>Very hard effort 4	C14
Do you have experience of migrant work?<br>[Defined as staying far from home for seasonal work]	Yes *1*<br>No 2	MIGR
Can you give an estimate of the monthly household income if I read some options to you? Is it<br>*[INSERT QUINTILE VALUES IN LOCAL CURRENCY]*<br>*(READ OPTIONS)*	≤ Quintile (Q) 1 1<br>More than Q 1, ≤ Q 2 2<br>More than Q 2, ≤ Q 3 3<br>More than Q 3, ≤ Q 4 4<br>More than Q 4 5<br>Don't Know 77<br>Refused 88	C15

For peer review only - http://bmjopen.bmj.com/site/about/guidelines.xhtml

CO-DEGREE: baseline questionnaire

PASTE THE ID LEVEL HERE

Participant Identification Number └─┴─┴─┘ └─┴─┴─┘ └─┴─┴─┘

During the past 12 months, how frequently have you had at least one standard alcoholic drink?<br>*(READ RESPONSES, USE SHOWCARD)*	Daily 1<br>5-6 days per week 2<br>3-4 days per week 3<br>1-2 days per week 4<br>1-3 days per month 5<br>Less than once a month 6<br>Not at all 7<br>Refused 88	A4
Do you currently smoke any tobacco products, such as cigarettes, cigars or pipes? *(USE SHOWCARD)*	Yes 1<br>No 2	*T1*
In a typical week, on how many days do you eat MEAT *(USE SHOWCARD)*	Number of days └─┴─┘<br>Don't Know 77	*D1*
Does your work involve vigorous-intensity activity that causes large increases in breathing or heart rate like *[carrying or lifting heavy loads, digging or construction work]* for at least 10 minutes continuously? *(OR USE SHOWCARD)*	Yes *1*<br>No 2	*P1*
Have you ever been told by a doctor or other health worker that you have raised blood pressure or hypertension?	Yes 1<br>No 2	H2a
Have you ever been told by a doctor or other health worker that you have raised blood sugar or diabetes?	Yes 1<br>No 2	H7a
Have you used agrichemicals?	Yes 1<br>No 2	L1
Did you mix, apply or both?	Mix 1<br>Apply 2<br>Both 3	L2
Have you been diagnosed with?	Dengue 1<br>Chikungunya 2<br>Zika 3<br>Malaria 4	L3
**Renal Protocol**		
Has a doctor diagnosed you with kidney disease?	No 1 go to question KI3<br>Yes 2 go to question KI2	KI1
Have you been told you have one of these kidney disease?	Glomerulonephritis 1<br>Congenital abnormality of the 2<br>kidneys 3<br>Polycystic kidney disease 4<br>Diabetic kidney disease 5<br>[locally defined] 6<br>[locally defined] 7<br>[locally defined] 8	KI2
Have you been told you have ever been told you have one of these diseases?	Tuberculosis 1<br>HIV 2<br>Hepatitis B 3<br>Hepatitis C 4<br>Schistosomiasis 5<br>Leptospirosis 6<br>[locally defined] 7<br>[locally defined] 8	KI3
Do you take herbal or traditional remedies?	No 1<br>Yes 2	KI4
Do you take regular prescribed medications?	No 1 go to question KI 9<br>Yes 2 2 go to questions below	KI5
Do you take medication for diabetes?	No 1<br>Yes 2	KI6
Do you take medication against HIV or hepatitis?	No 1<br>Yes 2	KI7
Do you take medication for tuberculosis?	No 1<br>Yes 2	KI8
Have you used painkillers most days for more than several months?<br>[Use Showcard with locally available medications]?	No 1<br>Yes 2	KI9

For peer review only - http://bmjopen.bmj.com/site/about/guidelines.xhtml

CO-DEGREE: baseline questionnaire

PASTE THE ID LEVEL HERE

Participant Identification Number └─┴─┴─┘ └─┴─┴─┘ └─┴─┴─┘

DEGREE study core lab measurements

Question	Response	Code
Blood sampling Investigator ID	└─┴─┴─┘	B2
Time of day blood specimen taken (24 hour clock)	Hours : minutes └─┘ : └─┘ hrs mins	B4
Creatinine measurement Technician ID	└─┴─┴─┘	CR1
Creatinine measurement Device ID	└─┴─┴─┘	CR2
Serum Creatinine	*to first decimal place if in mg/dL* └─┴─┴─┘	CR3
Serum Creatinine Units	mg/dL 1<br>µMol/L 2	CR4
Urine sampling Investigator ID	└─┴─┴─┘	UR1
Urinalysis Device ID	└─┴─┴─┘	UR2
Urine Glucose	Negative 1<br>100mg/dL 2<br>250mg/dL 3<br>500mg/dL 4<br>1000mg/dL 5<br>>2000mg/dL 6	UR3
Urine Specific Gravity	1.000 1<br>1.005 2<br>1.010 3<br>1.015 4<br>1.020 5<br>1.025 6<br>1.030 7	UR4
Urinalysis Blood	Negative 1<br>Non-haemolysed trace 2<br>Non-haemolysed moderate 3<br>Haemolysed trace 4<br>Small (+) 5<br>Moderate (++) 6<br>Large (+++) 7	UR5
Urine pH	5.0 1<br>6.0 2<br>6.5 3<br>7.0 4<br>7.5 5<br>8.0 6<br>8.5 7	UR6
Urinalysis Protein	Negative 1<br>Trace 2<br>30mg/dL (+) 3<br>100mg/dL (++) 4<br>300mg/dL (+++) 5<br>>2000mg/dL 6	UR7
Urinalysis Nitrite	Negative 1<br>Positive 2	UR8
Urinalysis Leucocytes	Negative 1<br>Trace 2<br>Small (+) 3<br>Moderate (++) 4<br>Large (+++) 5	UR9

For peer review only

For peer review only - http://bmjopen.bmj.com/site/about/guidelines.xhtml

CO-DEGREE: follow-up questionnaire

PASTE THE ID LEVEL HERE

Participant Identification Number |__|__|__| |__|__|__| |__|__|__|

CO-DEGREE Basic Core Questionnaire

	Response	Code
Study site ID	|__|__|__|	I1
Interviewer ID	|__|__|__|	I2
Study visit number	|__|__|__|	I3
Date of completion of the instrument	|__|__| dd |__|__| mm |__|__|__|__| year	I4

Interview Language and Name	Response	Code
Interview Language *[Insert Language]*	English 1<br>*[Add others]* 2<br>*[Add others]* 3<br>*[Add others]* 4	I6
Time of interview<br>(24 hour clock)	|__|__| : |__|__| hrs mins	I7
Family Surname		I8
First Name		I9
Address:		
Additional Information that may be helpful		
Contact phone number where possible		I10

For peer review only - http://bmjopen.bmj.com/site/about/guidelines.xhtml

CO-DEGREE: follow-up questionnaire

PASTE THE ID LEVEL HERE

Participant Identification Number |__|__|__| |__|__|__| |__|__|__|

CO-DEGREE basic core clinical and anthropometry measurements

Question	Response	Code
Ambient Temperature<br>(at time of examination measured in shade)	|__|__|__| °C	Temp
**Blood Pressure**		
Question	Response	Code
Interviewer ID	|__|__|__|	M1
Device ID for blood pressure	|__|__|__|	M2
Cuff size used	Small 1<br>Medium 2<br>Large 3	M3
Reading 1	Systolic (mmHg) |__|__|__|	M4a
	Diastolic (mmHg) |__|__|__|	M4b
	Heart rate |__|__|__|	M4c
Reading 2	Systolic (mmHg) |__|__|__|	M5a
	Diastolic (mmHg) |__|__|__|	M5b
	Heart rate |__|__|__|	M5c
Reading 3	Systolic (mmHg) |__|__|__|	M6a
	Diastolic (mmHg) |__|__|__|	M6b
	Heart rate |__|__|__|	M6c
During the past two weeks, have you been treated for raised blood pressure with drugs (medication) prescribed by a doctor or other health worker?	Yes 1<br>No 2	M7
**Height, and Weight**		
For women: Are you pregnant?	Yes 1<br>No 2	M8
Have you eaten yet today?	Yes 1<br>No 2	M9
Interviewer ID	|__|__|__|	M10
Height	in Centimetres (cm) |__|__|__|	M11
Weight<br>*If too large for scale 666.6*	in Kilograms (kg) |__|__|__|	M12

For peer review only - http://bmjopen.bmj.com/site/about/guidelines.xhtml

CO-DEGREE: follow-up questionnaire

PASTE THE ID LEVEL HERE

Participant Identification Number |__|__|__| |__|__|__| |__|__|__|

CO-DEGREE basic core questionnaire (adapted from DEGREE PROTOCOL)

Question	Response	Code
Sex (*Record Male / Female as observed*)	Male 1<br>Female 2	C1
How old are you?	Years |__|__|__|	C3
Which of the following best describes your main work status over the past 12 months?	Government employee 1<br>Non-government employee 2<br>Self-employed 3<br>Non-paid 4<br>Student 5<br>Homemaker 6<br>Retired 7<br>Unemployed (able to work) 8<br>Unemployed (unable to work) 9<br>Unpaid domestic 10<br>Refused 88	C8
If you are working what is your main occupation [FREE TEXT]:		OCCTXT
What task do you perform? [FREE TEXT]		TASKTXT
How many years have you been working in your current job?	Years |__|__|__|	C9
How many hours do you work daily?	Hours |__|__|__|	C10
Where do you work mostly?	Indoors 1<br>Outdoors 2<br>Both 3	C9
Do you take work breaks in shade?	Yes 1<br>No 2	C10
Do you work in a very hot working environment?	Seldom or never 1<br>Few times 2<br>Regularly 3<br>Frequently 4<br>Always or almost always 5	C11
How much physical effort did you do at work?	Slight effort 1<br>Moderate effort 2<br>Hard effort 3<br>Very hard effort 4	C12
Do you have experience of migrant work?<br>[Defined as staying far from home for seasonal work]	Yes *1*<br>No 2	MIGR
Can you give an estimate of the monthly household income if I read some options to you? Is it<br>*[INSERT QUINTILE VALUES IN LOCAL CURRENCY]*<br><br>*(READ OPTIONS)*	≤ Quintile (Q) 1 1<br>More than Q 1, ≤ Q 2 2<br>More than Q 2, ≤ Q 3 3<br>More than Q 3, ≤ Q 4 4<br>More than Q 4 5<br>Don't Know 77<br>Refused 88	C13
During the past 12 months, how frequently have you had at least one standard alcoholic drink?<br><br>*(READ RESPONSES, USE SHOWCARD)*	Daily 1<br>5-6 days per week 2<br>3-4 days per week 3<br>1-2 days per week 4<br>1-3 days per month 5<br>Less than once a month 6<br>Not at all 7<br>Refused 88	A4

For peer review only - http://bmjopen.bmj.com/site/about/guidelines.xhtml

CO-DEGREE: follow-up questionnaire

PASTE THE ID LEVEL HERE

Participant Identification Number |__|__|__| |__|__|__| |__|__|__|

Do you currently smoke any tobacco products, such as cigarettes, cigars or pipes? *(USE SHOWCARD)*	Yes 1<br>No 2	*T1*
In a typical week, on how many days do you eat MEAT *(USE SHOWCARD)*	Number of days<br>Don't Know 77 |__|__|	*D1*
Does your work involve vigorous-intensity activity that causes large increases in breathing or heart rate like *[carrying or lifting heavy loads, digging or construction work]* for at least 10 minutes continuously? *(OR USE SHOWCARD)*	Yes *1*<br>No 2	*P1*
Have you ever been told by a doctor or other health worker that you have raised blood pressure or hypertension?	Yes 1<br>No 2	H2a
Have you ever been told by a doctor or other health worker that you have raised blood sugar or diabetes?	Yes 1<br>No 2	H7a
Have you used agrichemicals?	Yes 1<br>No 2	L1
Did you mix, apply or both?	Mix 1<br>Apply 2<br>Both 3	L2
Have you been diagnosed with?	Dengue 1<br>Chikungunya 2<br>Zika 3<br>Malaria 4	L3
**Renal Protocol**		
Has a doctor diagnosed you with kidney disease?	No 1 go to question KI3<br>Yes 2 go to question KI2	KI1
Have you been told you have one of these kidney disease?	Glomerulonephritis 1<br>Congenital abnormality of the 2<br>kidneys 3<br>Polycystic kidney disease 4<br>Diabetic kidney disease 5<br>[locally defined] 6<br>[locally defined] 7<br>[locally defined] 8	KI2
Have you been told you have ever been told you have one of these diseases?	Tuberculosis 1<br>HIV 2<br>Hepatitis B 3<br>Hepatitis C 4<br>Schistosomiasis 5<br>Leptospirosis 6<br>[locally defined] 7<br>[locally defined] 8	KI3
Do you take herbal or traditional remedies?	No 1<br>Yes 2	KI4
Do you take regular prescribed medications?	No 1 go to question KI 9<br>Yes 2 2 go to questions below	KI5
Do you take medication for diabetes?	No 1<br>Yes 2	KI6
Do you take medication against HIV or hepatitis?	No 1<br>Yes 2	KI7
Do you take medication for tuberculosis?	No 1<br>Yes 2	KI8
Have you used painkillers most days for more than several months?<br>[Use Showcard with locally available medications]?	No 1<br>Yes 2	KI9

For peer review only - http://bmjopen.bmj.com/site/about/guidelines.xhtml

CO-DEGREE: follow-up questionnaire

PASTE THE ID LEVEL HERE

Participant Identification Number └─┴─┴─┘ └─┴─┴─┘ └─┴─┴─┘

DEGREE study core lab measurements

Question	Response	Code
Blood sampling Investigator ID	└─┴─┴─┘	B2
Time of day blood specimen taken (24 hour clock)	Hours : minutes └─┴─┘ : └─┴─┘ hrs mins	B4
Creatinine measurement Technician ID	└─┴─┴─┘	CR1
Creatinine measurement Device ID	└─┴─┴─┘	CR2
Serum Creatinine	*to first decimal place if in mg/dL* └─┴─┴─┘	CR3
Serum Creatinine Units	mg/dL 1<br>μMol/L 2	CR4
Urine sampling Investigator ID	└─┴─┴─┘	UR1
Urinalysis Device ID	└─┴─┴─┘	UR2
Urine Glucose	Negative 1<br>100mg/dL 2<br>250mg/dL 3<br>500mg/dL 4<br>1000mg/dL 5<br>>2000mg/dL 6	UR3
Urine Specific Gravity	1.000 1<br>1.005 2<br>1.010 3<br>1.015 4<br>1.020 5<br>1.025 6<br>1.030 7	UR4
Urinalysis Blood	Negative 1<br>Non-haemolysed trace 2<br>Non-haemolysed moderate 3<br>Haemolysed trace 4<br>Small (+) 5<br>Moderate (++) 6<br>Large (+++) 7	UR5
Urine pH	5.0 1<br>6.0 2<br>6.5 3<br>7.0 4<br>7.5 5<br>8.0 6<br>8.5 7	UR6
Urinalysis Protein	Negative 1<br>Trace 2<br>30mg/dL (+) 3<br>100mg/dL (++) 4<br>300mg/dL (+++) 5<br>>2000mg/dL 6	UR7
Urinalysis Nitrite	Negative 1<br>Positive 2	UR8
Urinalysis Leucocytes	Negative 1<br>Trace 2<br>Small (+) 3<br>Moderate (++) 4<br>Large (+++) 5	UR9

For peer review only
